# Liver-targeted delivery of TSG-6 by calcium phosphate nanoparticles for the management of liver fibrosis

**DOI:** 10.7150/thno.37301

**Published:** 2020-01-01

**Authors:** Min Wang, Miao Zhang, Lianhua Fu, Jing Lin, Xinmin Zhou, Pinghong Zhou, Peng Huang, Hao Hu, Ying Han

**Affiliations:** 1 Department of Gastroenterology, Hepatology and Nutrition, Shanghai Children's Hospital, Shanghai Jiao Tong University, Shanghai 200062, China; 2 Xijing Hospital of Digestive Diseases, State Key Laboratory of Cancer Biology, Fourth Military Medical University, Xi'an 710032, China; 3 Marshall Laboratory of Biomedical Engineering, International Cancer Center, Laboratory of Evolutionary Theranostics (LET), School of Biomedical Engineering, Shenzhen University Health Science Center, Shenzhen, 518060, China; 4 Endoscopy Center of Zhongshan Hospital, Fudan University, Shanghai 200032, China

**Keywords:** Liver fibrosis, Tumor necrosis factor-stimulated gene 6 (TSG-6), Calcium phosphate nanoparticle, Liver targeting delivery, matrix metalloproteinase 12 (MMP12), bovine serum albumin (BSA)

## Abstract

Mesenchymal stem cells (MSCs) transplantation is a promising antifibrotic strategy but facing clinical controversies. Inspired by advances in nanomedicine, we aimed to bypass these clinical barriers of MSCs by identifying the key antifibrotic molecule of MSCs and developing a specific liver-targeting nanocarrier.

**Methods**: Cytokines secreted by MSCs were examined with serum stimulation of cirrhotic patients. Immunohistochemistry, microarray, immunoblotting, and quantitative real-time PCR (qRT-PCR) were applied to identify the critical antifibrotic cytokine and to discover its role in modulating antifibrotic effects. Biomineralization method was used to prepare calcium phosphate nanoparticles (NPs). The targeting and therapeutic efficiency of NPs were evaluated by *in vivo* imaging and biochemical studies on fibrotic mice induced by CCl_4_.

**Results**: The stimulated MSCs exhibited high-level expression of Tumor necrosis factor (TNF)-stimulated gene 6 (TSG-6). On animal study, exogenous administration of TSG-6 alone can ameliorate liver fibrosis while TSG-6 knocked MSCs (Lv-TSG-6 MSCs) lost antifibrotic effects. Further studies verified the importance of TSG-6 and identified its antifibrotic mechanism by modulating M2 macrophages and increasing matrix metalloproteinase 12 (MMP12) expression. Additionally, we found a feedback loop between TSG-6, MMP12 and pro-inflammatory cytokines (TNF-α, IL-6, and IL-1β), which may improve our understanding of the aggravating process of cirrhosis and antifibrotic mechanisms of TSG-6 and MSCs. Based on these findings, we developed calcium phosphate nanoparticles (CaP@BSA NPs) by biomineralization method using bovine serum albumin (BSA) as the biotemplate. Imaging tracking and drug loading studies showed specific liver targeting and high TSG-6 loading efficacy of as-prepared CaP@BSA NPs. *In vivo* therapeutic study further demonstrated the improved therapeutic effects of TSG-6 loaded CaP@BSA.

**Conclusions**: TSG-6 was a major antifibrotic cytokine of MSCs, TSG-6 loaded CaP@BSA NPs showed specific liver accumulation and improved therapeutic effects, which indicated translational potentials of CaP@BSA as a promising drug carrier for the liver disease management.

## Introduction

There is an increasing global burden of liver cirrhosis, which affected an estimated 2.8 million people and resulted in 1.3 million deaths each year 1, 2. Although liver transplantation is the only definitive treatment for end-stage cirrhosis, the shortage of transplantable organs and lifelong immunosuppression create the necessity for scientists and doctors to look for new antifibrotic and pro-regenerative therapies.

Mesenchymal stem cells (MSCs) transplantation is one of the most widely tested alternative strategies 3. Several clinical trials have shown MSCs contribution to liver regeneration and function restoration 4-10. However, MSCs transplantation is still a controversial field. One of the reasons is that the differentiation direction of MSCs is hardly controlled *in vivo.* These cells would differentiate into not only hepatocytes but myofibroblasts as well, and that means they may potentially deposit scars. Besides, cirrhotic patients are actually not suitable for autologous translation, but allogeneic MSCs would bring possible undesirable immune rejection and virus-carry engraftment. Currently, increasing evidence described MSCs as a “hit and run” therapy 11. MSCs display their therapeutic action by trophic mechanisms rather than replenishing injured tissue 12-15. MSCs secrete many kinds of bioactive molecules with tissue repair activities. Therefore, by identifying specific antifibrotic molecules and directly using these effectors instead 16, we could avoid cell transplantation and bypass above barriers of MSCs. However, targeting delivery of these active molecules to the fibrotic liver is a big challenge.

Excitingly, nanoparticles (NPs) are easily captured by the reticuloendothelial system (RES) of the liver 17. Calcium phosphate (CaP) is one kind of calcium-based biomaterials, which has gained special interests in biomedical fields due to its excellent biocompatibility, bioactivity, and biodegradability 18. Under physiological conditions, CaP is relatively stable, thus can effectively avoid the premature leakage of loaded drugs. Besides, the Ca^2+^ and PO_4_^3-^ ions can participate in the normal metabolism of organisms, which can overcome the dilemmas including poor biodegradability and potential long-term toxicity of traditional inorganic biomaterials such as silica-, carbon-, and gold-based biomaterials. CaP NPs has been demonstrated as an excellent drug delivery system. For instance, Han *et al.* developed biostable CaPs NPs with the high tumor-targeting ability through the PEG-conjugated hyaluronic acid-involved mineralization 19, 20. Zhang *et al.* constructed a multifunctional spherical polydopamine/ mesoporous CaP NPs with hollow cavities served as storage spaces and passages for the anti-cancer drug, doxorubicin (DOX) 21. However, there is no published paper reporting application of CaP NPs for liver disease drug delivery. It's worth noting that the physiological and biological characteristics of CaP can be easily modified by using different biotemplates, such as proteins and peptides 22 and BSA is a commonly used biotemplate that has been suggested to increase liver accumulation 23-25. Therefore, adopting BSA as a biotemplate to develop CaP NPs may lead to the development of novel liver targeting drug nanocarrier.

In this study, TSG-6 was identified as pivotal antifibrosis cytokine of MSCs, which showed strong antifibrotic activity. TSG-6 injection alone was as effective as MSCs transplantation. TSG-6 induced M2 macrophage polarization and increased MMP12 expression. Importantly, we found an inhibitory loop between MMP12 and pro-inflammatory cytokines and discovered an MMP12 rescue effect of TSG-6, which may partly explain the aggravating process of cirrhosis and antifibrotic mechanisms of TSG-6 and MSCs. Afterward, we prepared CaP NPs by biomineralization method using BSA as the biotemplate (CaP@BSA). *In vivo* imaging and therapeutic studies showed high liver accumulation and improved therapeutic effects of TSG-6 loaded CaP@BSA NPs, which indicated the translational potential for the management of liver diseases.

## Methods

### Animals and experiment groups

The male C57BL/6J mice (8-10 weeks) were brought from the experimental animal center of Slaccas (Shanghai, China). All animals were cared in pathogen-free airflow cabinet and allowed free access to food and water. The animal study protocol was approved by the Animal Welfare and Ethics Committee of the Fourth Military Medical University (FMMU) and performed according to the “Guidelines for the Care and Use of Laboratory Animals”.

Mice fibrosis model was established using an intraperitoneal injection of 0.75 mL/kg CCL_4_ dissolved in olive oil twice a week. One day after 8 weeks injection, the fibrotic mice were randomly divided into groups (n = 6-7) receiving either PBS, 1 × 10^6^ differentially treated MSCs once (MSCs, TSG-6 knockdown MSCs and the corresponding negative control: Lv-shTSG-6 MSCs and Lv-nc MSCs, GFP^+^ MSCs, Dir-labeled MSCs) or three doses of 5 *μ*g recombinant human TSG-6 (2104-TS, R&D Systems, MN), CaP@BSA-TSG-6 on alternate days by tail vein injection. The mice were continually injected with CCl_4_ for another four weeks. One group devoid of CCL_4_ were used as control. Mice were collected blood from inner canthus or sacrificed for further analyses. In some cases, after Dir-labeled MSCs transplantation, mice were anesthetized to imaging *in vivo*. The bile duct ligation (BDL) operation was performed routinely. Acute liver injury (ALF) was induced by intraperitoneal administration 30 ng/g body weight of LPS and 150 *μ*L clodronate liposomes was intravenously injected 2 days prior to LPS to deplete *in vivo* macrophages. Then mice were sacrificed for analysis at 48 h after TSG-6 injection.

### Preparation of CaP@BSA NPs

CaP@BSA NPs were prepared according to the previous report with modification 26. Briefly, 10 mg BSA was dissolved in 1 mL Dulbecco's Modified Eagle medium (DMEM), sealed, and incubated at 37 °C for 24 h to reach equilibrium. Then, 10 *μ*L CaCl_2_ (1 M) was added into the reaction system for another 24 h. After incubation, the product was separated by centrifugation (13,500 rpm, 15 min), washed by deionized water for several times, freeze-dried and stored at -20 °C for further use.

### Statistical analysis

Quantitative data are presented as mean ± SD. Statistical significance between groups was determined using one-way ANOVAs with post hoc multiple comparisons or Student's *t*-tests if appropriate. Mann-Whitney *U* test was performed when parametric testing was not applicable. P values of < 0.05 were considered statistically significant. Statistical analysis was plotted by GraphPad Prism 7.0 (GraphPad Software, CA).

For further details regarding the materials used, please refer to the Supplementary Materials.

## Results

### MSCs are activated to express high-level TSG-6 *in vitro* and* in vivo*

**Figure 1:**
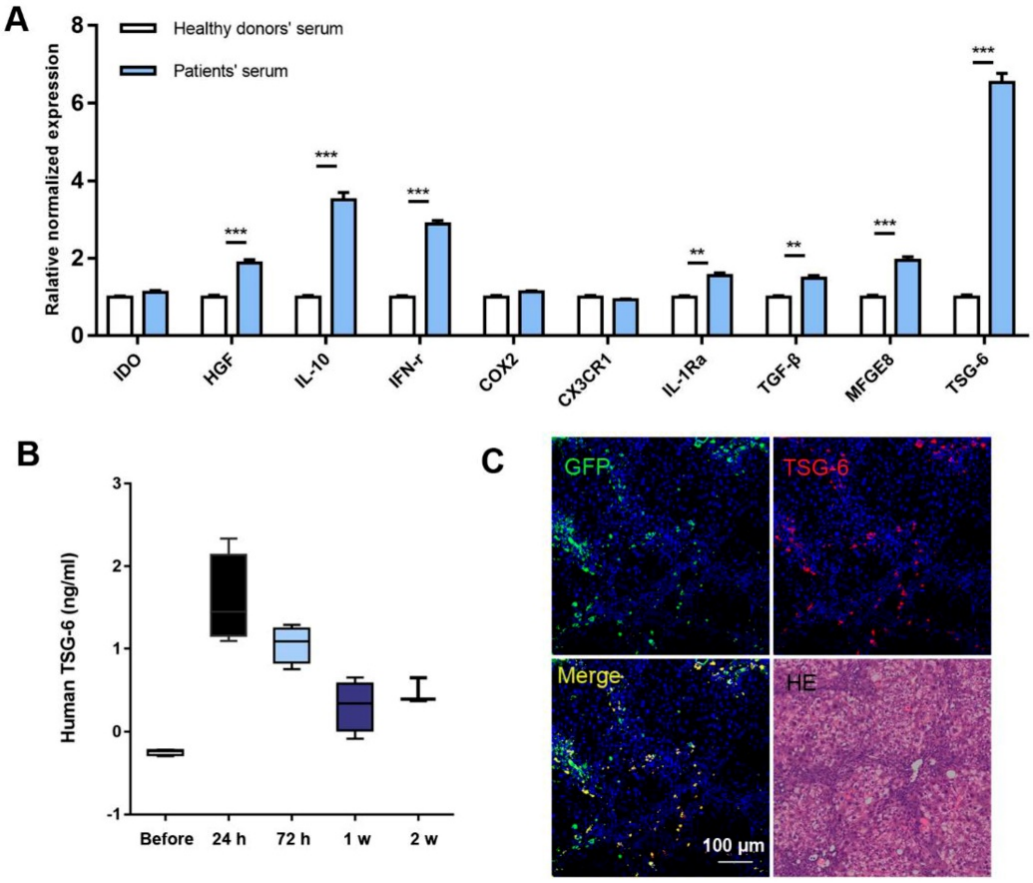
** MSCs secreted high level of TSG-*6 in vitro* and* in vivo*.** A) qRT-PCR analysis of 10 cytokines expression after serum stimulation of MSCs, those of healthy volunteers are referred to as 1. B) Serum TSG-6 level measured by ELISA at the indicated time (n = 4). C) The IF staining of mice liver using anti-TSG-6 antibody (red) and DAPI (blue) at 24 h after GFP^+^ MSCs (green) transplantation. Scale bars, 100 μm. *P < 0.05, **P < 0.01, ***P < 0.001.

The characters of MSCs were double confirmed by both flow-cytometry and differentiation assay (Fig. S1). We chose the 10 most reported therapeutic cytokines for comparing and evaluation. Serum samples from 5 cirrhotic patients were pooled for the preparation of MSCs stimulation medium and serum from 5 healthy volunteers were used as control. The detailed clinical information of the patients was listed in Table S1 and written informed consent was obtained from each individual. After 24 h stimulation, 7 of 10 cytokines showed significant increases compared to the controls, of which TSG-6 exhibited the highest fold increase compared to others. (6.52 ± 0.24) (Fig. 1A).

*In vivo* studies on fibrotic mice model also showed increased expression of TSG-6 after MSCs transplantation. At 24 h, serum TSG-6 reached peak concentration (Fig. 1B). Interestingly, the TSG-6 expression pattern was consistent with the* in vivo* behavior of MSCs: both near-infrared (NIR) fluorescence imaging (tracking Dir-labeled MSCs *in vivo*) and immunofluorescent (IF) staining (locating GFP^+^ MSCs) showed maximum hepatic engraftation happened at 24 h post-transplantation (Fig. S2). Co-localization of TSG-6 and engrafted MSCs indicated TSG-6 was secreted by the engraft MSCs (Fig. 1C).

### TSG-6 is indispensable for MSCs in attenuating liver cirrhosis

**Figure 2:**
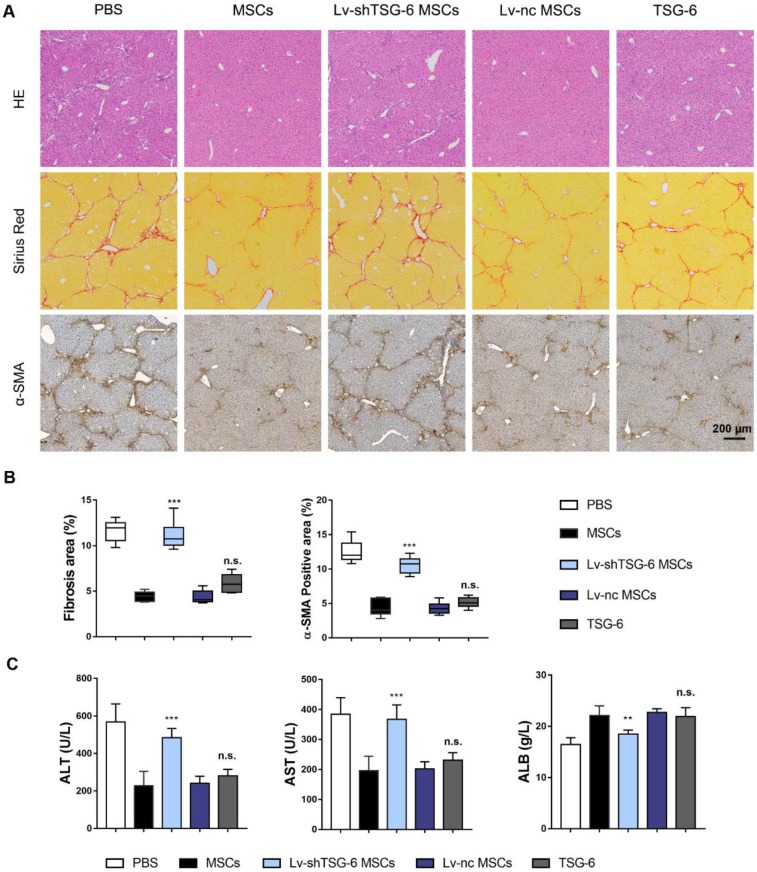
** Exogenous administration of TSG-6 is comparable with MSCs transplantation in ameliorating CCl_4_ induced liver fibrosis.** A) Representative histological images 4 weeks after MSCs, Lv-shTSG-6 MSCs, Lv-nc MSCs or TSG-6 treatment. HE staining (top); Sirius red (middle); α-SMA (bottom). Scale bars, 200 μm; B) Quantification of fibrotic (Sirius red) and α-SMA^+^ areas; C) Changes of serum ALT, AST, and ALB in different groups. n = 6 for each group. n.s.: non-significance, **P < 0.01, ***P < 0.001 compared to MSCs group. Percentages were measured by Image-Pro Plus.

To further assess the importance of TSG-6 in attenuating liver fibrosis, we established TSG-6 knockdown MSCs (Lv-shTSG-6 MSCs). The knockdown efficiency was shown in Fig. S3. And then, MSCs, Lv-shTSG-6 MSCs, scramble shRNA transfected MSCs (Lv-nc MSCs) and human recombinant TSG-6 were intravenously injected into fibrotic mice respectively. The therapeutic evaluation was carried out 4 weeks later. Results showed that the degree of collagen deposition and area of hepatic necrosis were significantly reduced by administration of MSCs and Lv-nc MSCs compared to that of Lv-shTSG-6 MSCs and PBS (P < 0.001, Fig. 2A, B). The α-SMA, a marker of pathologic fibroblasts, showed a significant decrease in MSCs and Lv-nc MSCs than that of TSG-6 knockdown MSCs (*P*<0.001, Fig. 2A, B). Importantly, the TSG-6 administration exhibited comparable anti-fibrotic effects as MSCs transplantation (Fig. 2A, B). The serum alanine aminotransferase (ALT), aspartate aminotransferase (AST), and albumin (ALB) tests indicated better hepatic function restoration by TSG-6 and MSCs treatment than Lv-shTSG-6 MSCs and PBS (P < 0.001, Fig. 2C). On another fibrotic mode, bile duct ligation (BDL) model, similar therapeutic effects of TSG-6 and MSC were also demonstrated as expected (Fig. S4). These findings suggested the importance of TSG-6 for MSCs and knockdown TSG-6 caused damages to their repair capacity, and exogenous TSG-6 administration was as effective as MSCs transplantation.

### Transcriptome analysis identified MMP12 was an important effector of TSG-6

**Figure 3:**
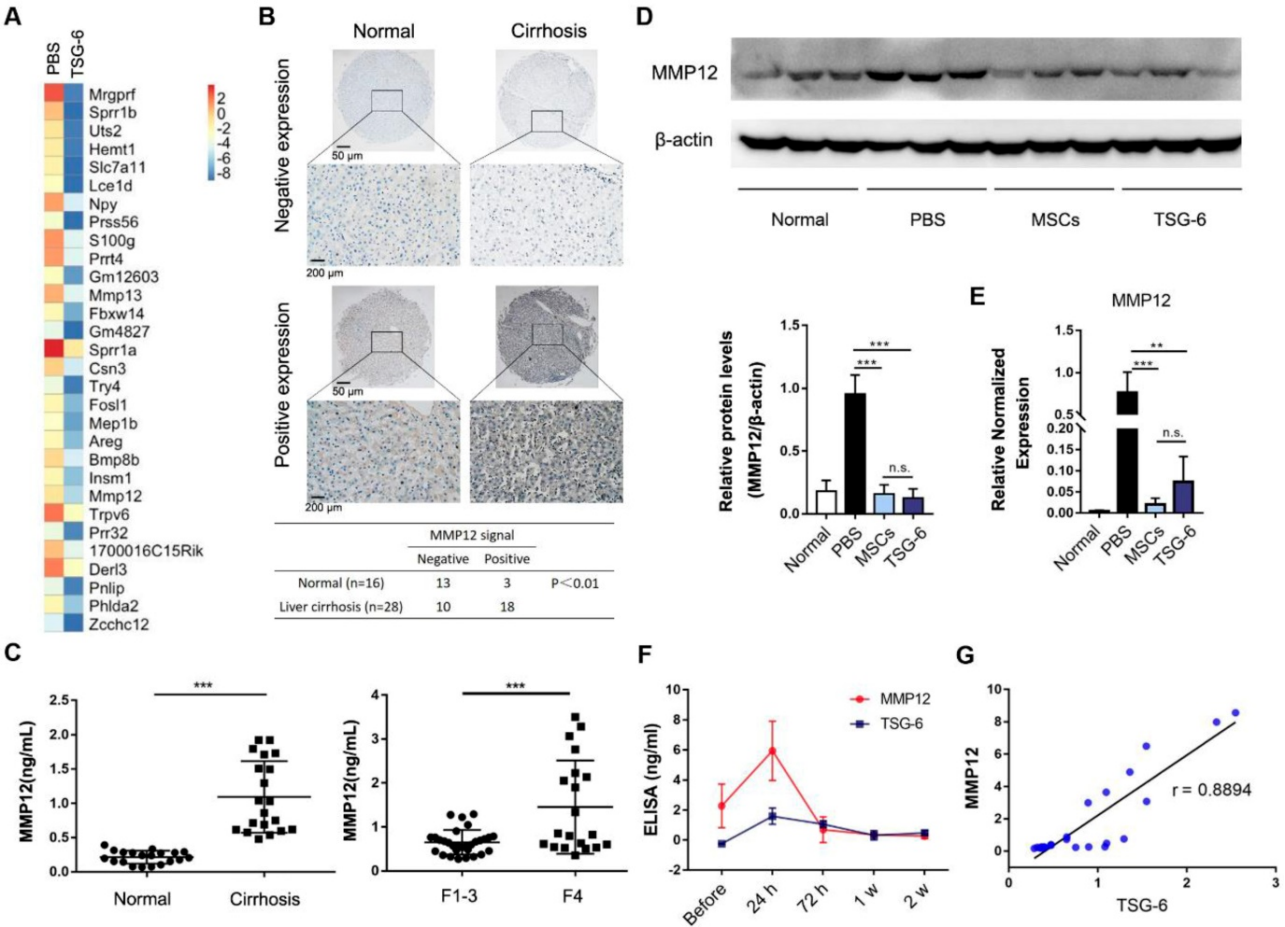
** MMP12 was an important effector of TSG-6.** A) Heatmap diagram of the top 30 down differentially expressed gene after TSG-6 treatment. Gene names appear in rows in row. Red bar indicated higher expression, and blue bar indicated lower expression. B) Expression of MMP12 in normal and cirrhotic patients. Scale bar 50 *μ*m and 200 *μ*m. C) Left: Serum MMP12 level in hepatitis B virus-related cirrhotic patients (n = 20) and normal (n = 20); Right: Serum MMP12 level in primary biliary cirrhosis patients with different fibrotic stages. F1-3 with no evident cirrhosis (n = 30), F4 with definite cirrhosis (n = 20); Fibrotic stages were evaluated according to the METAVIR scoring system. D) Western blot (upper) and quantitation analysis (lower) of mice hepatic MMP12 after treatment of MSCs and TSG-6 (n = 3). E) qRT-PCR analysis of mice hepatic MMP12 after treatment of MSCs and TSG-6 (n = 3). F) Serum dynamic changes of MMP12 and TSG-6 after MSCs transplantation (n = 6). G) linear correlation analysis between TSG-6 and MMP12 level. Each point represents an (x, y) pair of MMP12 and TSG-6 (n = 24). **P < 0.01, ***P < 0.001.

In order to further explore the therapeutic mechanism of TSG-6, liver transcriptome changes were analyzed by Agilent Gene Expression Microarray (mouse V2 8x60K). The microarray identified 320 upregulated and 412 downregulated mouse transcripts with a 2-fold or higher variation 2 weeks after TSG-6 treatment (Fig. 3A and Table S2). Meanwhile, TSG-6 failed to improve acute liver injury after depleting *in vivo* macrophages with clodronate liposomes, which indicated the necessity and importance of macrophages for TSG-6 antifibrotic effects (Fig. S5). Therefore, the macrophages related transcripts were subjectively examined for candidate genes of interest. MMP12, a member of matrix-degrading metalloproteinases, was greatly downregulated after TSG-6 treatment (Fig. 3A, fold change (FC) = 0.019). MMP12 attracted our attention since fibrosis is associated with the imbalance decomposition of the extracellular matrix, and more importantly, macrophages are reported to be its sole source 27. To our surprise, MMP12 levels showed to be relevant to the clinical fibrosis severity status. On tissue array, MMP12 expression was higher on cirrhotic livers than the normal (χ2 = 8.462; *P* < 0.01, Fig. 3B and Table S3). The positive and negative controls were shown in Fig. S6. Similarly, serum MMP12 level also exhibited significant increases in patients with cirrhosis and F4 fibrotic stage (Fig. 3C, *P* < 0.001, and Table S4-5). On the fibrotic mice model, similar MMP12 changes were also observed. Liver injury caused by CCl_4_ significantly increased MMP12 expression, and additional TSG-6 and MSCs treatment decreased MMP12 levels in liver (Fig. 3D, E). Time-dependent ELISA assay showed serum MMP12 changed with the level of TSG-6 with a median correlation index of *r* = 0.8894 (Fig. 3F, G). These results implied the importance of MMP12 and possible MMP12 regulatory roles of TSG-6.

**Figure 4:**
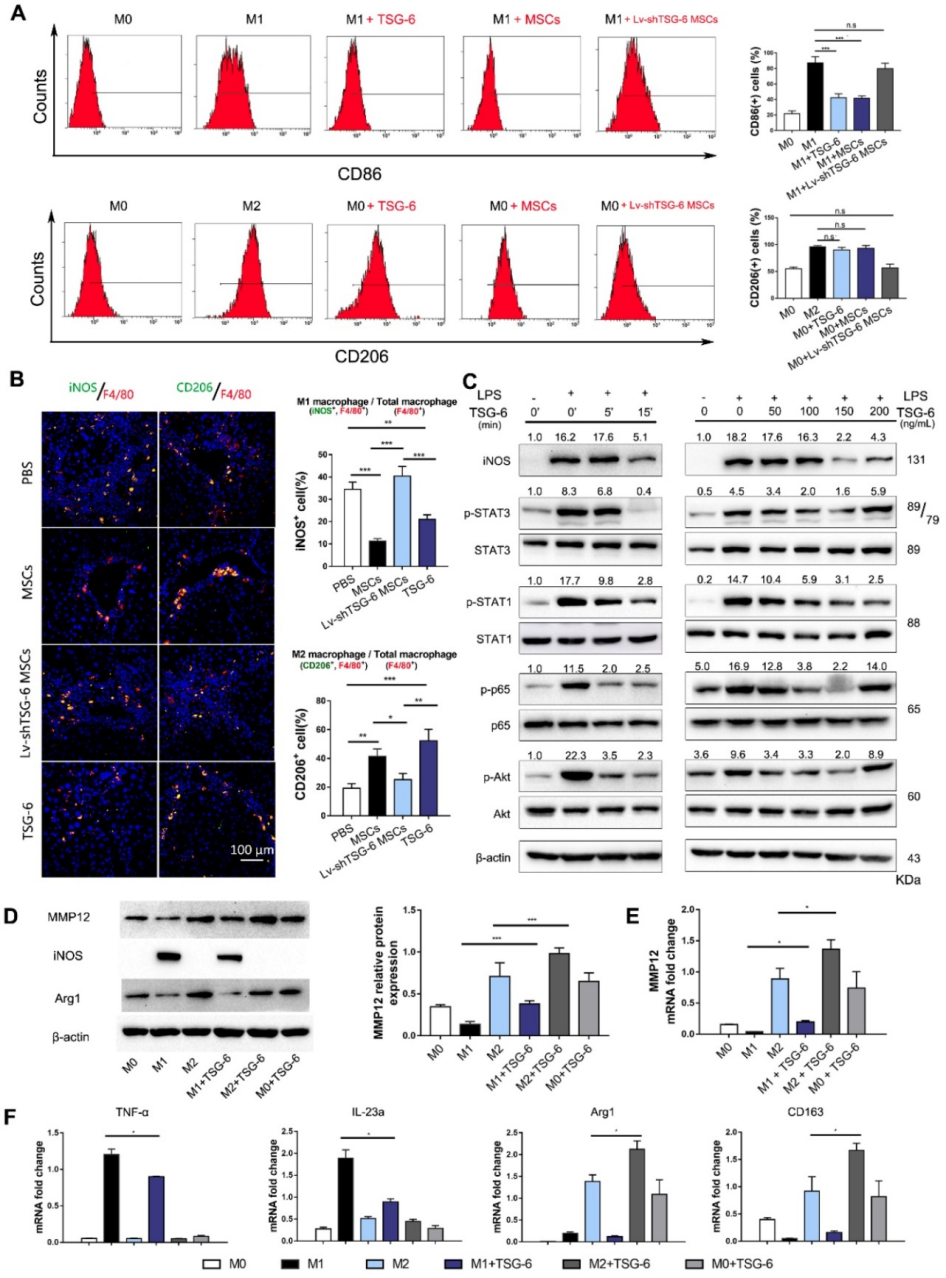
** TSG-6 promotes MMP12 expression by inducing M2 macrophages polarization.** A) Flow cytometry plots showing CD86 (M1 marker, upper) and CD206 (M2 marker, lower) changed with different stimulations. The percentage of CD86^+^ or CD206^+^ cells was shown in the histogram. B) Representative images showed the CD206^+^ M2 macrophages (green) and iNOS^+^ M1 macrophages (green) in each group. The nuclei were stained with DAPI and macrophages were stained with F4/80 (red). Scale bar, 100 *μ*m. The percentage of iNOS^+^ or CD206^+^ cells was calculated by Image-Pro Plus. C) TSG-6 dephosphorylated STAT3, STAT1, p65, and Akt. Quantified results are shown as the ratio of phosphorylated to total protein (Bottom). D) Western blot and quantification of MMP12 in groups receiving different treatments. E) mRNA changes of MMP12 in groups receiving different treatments. F) TSG-6 counterbalanced LPS/IFN-γ induction and promoted M2 transition. M1 marker (iNOS, TNF-α, IL-23a) and M2 marker (Arg1, CD163) were determined by qRT-PCR reactions. n.s.: non-significance, *P < 0.05, **P < 0.01, *** P < 0.001.

Based on the above findings, we hypothesize that TSG-6 and MSCs may control MMP12 through macrophages. To explore the effects of TSG-6 and MSCs on macrophage phenotypes, bone marrow-derived macrophages were isolated (Fig. S7) and classic M1/M2 phenotypes were established by LPS/IFN-γ and IL-4 stimulation. Flow cytometry showed that CD86^+^ cells, an M1 macrophage marker, significantly decreased to 43.5% and 41.6% due to TSG-6 and MSCs treatment (*P* < 0.001; Fig. 4A). However, a similar decline was not observed by Lv-shTSG6 MSCs infusion, in which 81.1% of CD86^+^ cells showed no significant difference with classic LPS/IFN-γ M1 stimulation (82.8%). In the meantime, both TSG-6 and MSCs showed to promote M2 transition and increased CD206^+^ cells to 90.5% and 93.2%, which is equivalent to classic IL-4 stimulation (96.8%, *P* > 0.05, Fig. 4A). As expected, Lv-shTSG-6 MSCs treatment did not increase CD206^+^ cells (59.7% vs. 58.8%, *P* > 0.05, Fig. 4A). Further *in vivo* experiment demonstrated that TSG-6 and MSCs increased liver M2 ratio (CD206^+^/F_4/80_) and decreased M1 ratio (iNOS^+^/F_4/80_) when compared with PBS and Lv-shTSG-6 MSCs (Fig. 4B). Moreover, hepatic macrophages isolated from TSG-6 treated mice exhibited M2 functions, showing reduced production of TNF-α, IL-6, IFN-γ, and IL-12p70 but increased the production of IL-10 and IL-4 (Fig. S8). These data suggested TSG-6 and MSCs not only increase M2 transition but importantly make these cells function with anti-inflammatory effects. Pathway analysis revealed that TSG-6 inhibited phosphorylation of STAT1, STAT3, p65, and Akt, all of which were involved in M2 polarization in a time-dependent and dose-dependent manner (Fig. 4C). TSG-6 also blocked nuclear translocation of NF-κB in M1 macrophages (Fig. S9), which impaired NF-κB associated inflammatory cascades and restricted the pro-inflammatory effects of M1 cells.

Next, we compared the MMP12 expression difference between M1 and M2 macrophages. MMP12 was found mainly expressed in M2 macrophages, and IL-4 stimulation induced higher MMP12 expression than LPS/IFN-γ treated and parental M0 macrophages (Fig. 4D, E). In addition, TSG-6 could further increase M2 cells to express more MMP12 and counterbalance against LPS/IFN-γ forced M1 transition, with increased M2 markers but decreased M1 markers (Fig. 4D-F).

### MMP12 suppressed HSCs activation and pro-inflammatory cytokines release

**Figure 5:**
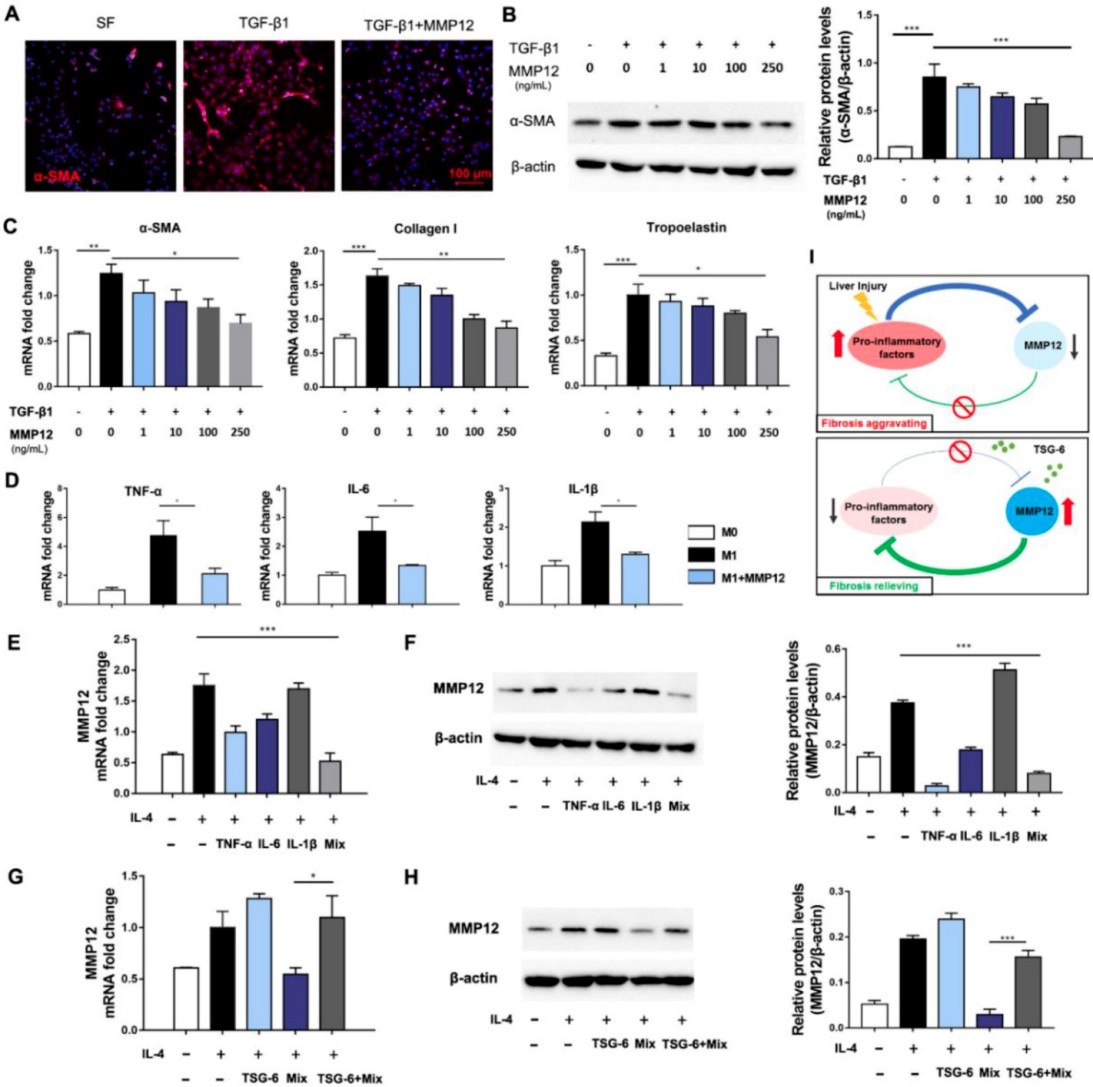
** MMP12 suppressed HSCs activation and pro-inflammatory factors release.** A) Representative IF images of HSCs that treated with TGF-β1 with or without the presence of MMP12. α-SMA, red. Scale bars, 100 *μ*m. SF: serum-free. B) Left: MMP12 suppressed the α-SMA expression of HSCs in a dose-dependent manner; Right: quantification of MMP12 in groups receiving different treatments. C) MMP12 reduced mRNA of α-SMA, collagen I and tropoelastin in a dose-dependent manner. D) MMP12 suppressed M1 macrophages expressing TNF-α, IL-6, and IL-1β. E-F) TNF-α, IL-6, IL-1β and their mixture inhibited MMP12 expression. E: qRT-PCR; F: Western blot and quantification analysis. G-H) TSG-6 restored MMP12 expression even in the presence of pro-inflammatory cytokines. G: qRT-PCR; H: Western blot and quantification analysis. I) Schematic overview of the inhibitory loop with or without TSG-6 existence. *P < 0.05, **P < 0.01, ***P < 0.001.

Hepatic stellate cells (HSCs) were the primary source of extracellular matrix, and their activation is the key to the formation of hepatic fibrosis 28, 29. Next, we analyzed MMP12 effects on HSCs. Results showed that MMP12 suppressed human HSCs cell line, LX2, to express α-SMA in a dose-dependent manner (Fig. 5A-C). In the meantime, MMP12 inhibited collagen I and tropoelastin production as well, which are usually expressed by activated HSCs.

Moreover, MMP12 was found with inhibitory effects on M1 macrophages and restrained them from releasing pro-inflammatory cytokines, such as TNF-α, IL-6, and IL-1β (Fig. 5D). On the contrary, TNF-α, IL-6, IL-1β, and their combination conversely inhibited MMP12 expression. The mutual relationships between MMP12 and pro-inflammatory cytokines formed an inhibitory feedback loop (Fig. 5E, F). Interesting, we discovered that TSG-6 could interrupt this inhibitory loop and rescue MMP12 expression even in the presence of pro-inflammatory cytokines (Fig. 5G, H). This feedback loop may help our understanding of the self-aggravating process of liver fibrosis and the therapeutic mechanisms of MSCs and TSG-6 (Fig. 5I): Persisted inflammation produces a great number of inflammatory activators, which suppress MMP12 and its antifibrotic effects, leading fibrosis to progress. While exogenous TSG-6 or MSCs administration could interrupt such inhibition and restore MMP12 expression, which in-turn suppress HSCs activation and inflammatory cytokines release, therefore results in fibrosis alleviation.

### Synthesis and characterization of CaP@BSA NPs

Since most exogenous TSG-6 remained in blood circulation after intravenous injection, strategies to improve liver targeting and releasing are expected to enhance TSG-6 therapeutic effects. A suitable carrier to deliver TSG-6 to the liver and realize sustained-release simultaneously is highly desirable. Herein, the biodegradable and biocompatible CaP@BSA hollow NPs are prepared by a biomineralization method and evaluated as a delivery system for liver targeting and the controlled release of TSG-6.

**Figure 6:**
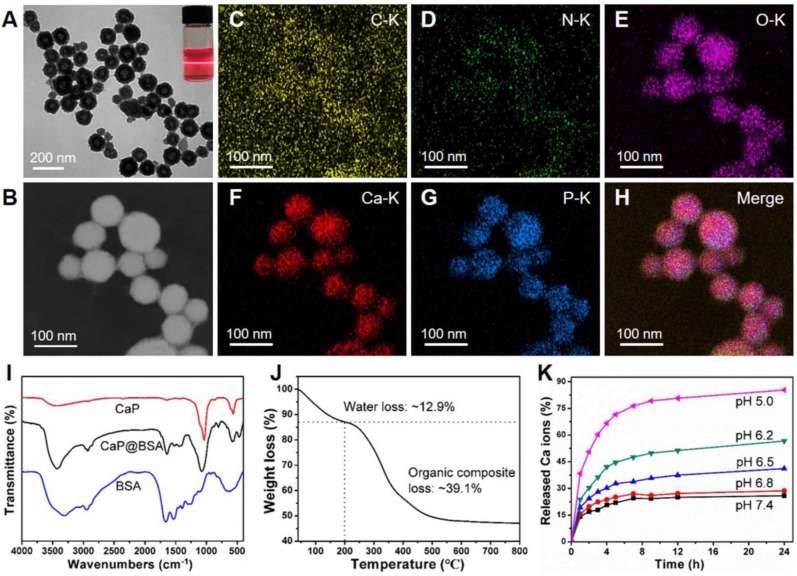
** Synthesis and characterization of CaP@BSA NPs.** A) The representative TEM image of the CaP@BSA NPs. Top right corner, the optical photograph of Tyndall scattering after Ca introduction in the reaction system. B-H) STEM image and elemental mapping analysis of CaP@BSA NPs. I) FT-IR spectra of CaP@BSA NPs, pure BSA, and bare CaP prepared in the absence of BSA. J) TG analysis of CaP@BSA NPs. K) The release property of CaP@BSA NPs in PBS buffer at different pH values.

The spherical CaP@BSA hollow NPs (Fig. 6A) with a coarse surface (Fig. S10) were obtained by using a mineralization process of BSA in a biomimetic medium (DMEM containing 1.8 mM of Ca^2+^ and 0.9 mM of PO_4_^3-^ ions). The average size and shell thickness of the NPs are 98 ± 24 nm and 37 ± 5 nm, respectively (Fig. S11). The obtained NPs with such size distribution are expected with good liver targeting since it has been demonstrated that the NPs encapsulated BSA ranged in 100 and 200 nm could serve as a rational liver-targeted drug delivery system 30. As a biomineralization-related protein, BSA was applied to interact with Ca^2+^ ions in DMEM to increase local supersaturation and provide nucleation sites. After the introduction of Ca^2+^ ions, the presence of NPs in the medium can be verified by obvious Tyndall scattering (the inset of Fig. 6A). The main elemental compositions of hollow NPs were determined to be C, N, O, Ca, P, suggesting CaP@BSA complex formation (Fig. 6B-H). The morphology of CaP@BSA NPs can be tuned by simply adjusting the BSA concentration (Fig. S12). In the absence of BSA, bare CaP crystals with irregular morphology were formed. With the increase of BSA concentration, the obtained NPs showed a morphology evolution from solid nanospheres to hollow NPs, suggesting that the organized structures came from BSA-mediated biomineralization. With uniform and well-defined hollow structures, CaP@BSA NPs prepared at a BSA concentration of 10 mg/mL were chosen for further experiments.

The fourier-transform infrared spectroscopy (FT-IR) spectra of products showed the characteristic peaks of BSA (1643 cm^-1^, C=O vibration) and CaP (1036 cm^-1^, P-O vibration), demonstrating the formation of CaP@BSA complexes (Fig. 6I). From the thermogravimetric analysis (TGA) (Fig. 6J), the content of water was around 12.9%, the organic components were accounted for around 39.1%, and the content of inorganic CaP was 48.0% of CaP@BSA NPs. The pH-responsive degradation of CaP@BSA NPs was evaluated in PBS with different pH values to simulate different biological environments (Fig. 6K). Within 24 h, 25.9% of the Ca^2+^ ions were released at pH 7.4, whereas more Ca^2+^ ions were released from NPs in acidic environments, discharging 28.6% at pH 6.8, 41.1% at pH 6.5, 56.6% at pH 6.2, and 85.3% at pH 5.0. The zeta potential of NPs was negatively charged in deionized water (Fig. S13), demonstrating that CaP@BSA NPs can be applied as carriers for various drugs through electrostatic attraction. In this study, the TSG-6 loading capacities of CaP@BSA NPs was determined by ELISA. The TSG-6 loading efficiency and loading capacity of CaP@BSA NPs were 96.7% and up to 120.9 *μ*g/mg (*μ*g drug per mg carrier), respectively, at an initial TSG-6 concentration of 125 *μ*g/mL. Importantly, the TSG-6-loaded CaP@BSA NPs showed a sustained drug release property, and less than 15% of the drug was released at pH 7.4 (Fig. S14), indicating that the drug can be well maintained during circulation in the body. For the biocompatibility and biosafety assessment of CaP@BSA NPs, the toxicity and hemolysis tests were carried out. The MTT assay showed that the CaP@BSA NPs have excellent biocompatibility and biosafety, since cell viability was above 95% at all tested concentrations of 10 - 400 *μ*g/mL of NPs (Fig. S15), and mice treated with excessive CaP@BSA (400 *μ*g) showed no obvious damages to major organs (Fig. S16). The hemolysis rates were almost lower than 2.1% at NPs concentrations of 25-800 *μ*g/mL (Fig. S17).

### Liver targeting and sustained release TSG-6 of CaP@BSA NPs

**Figure 7:**
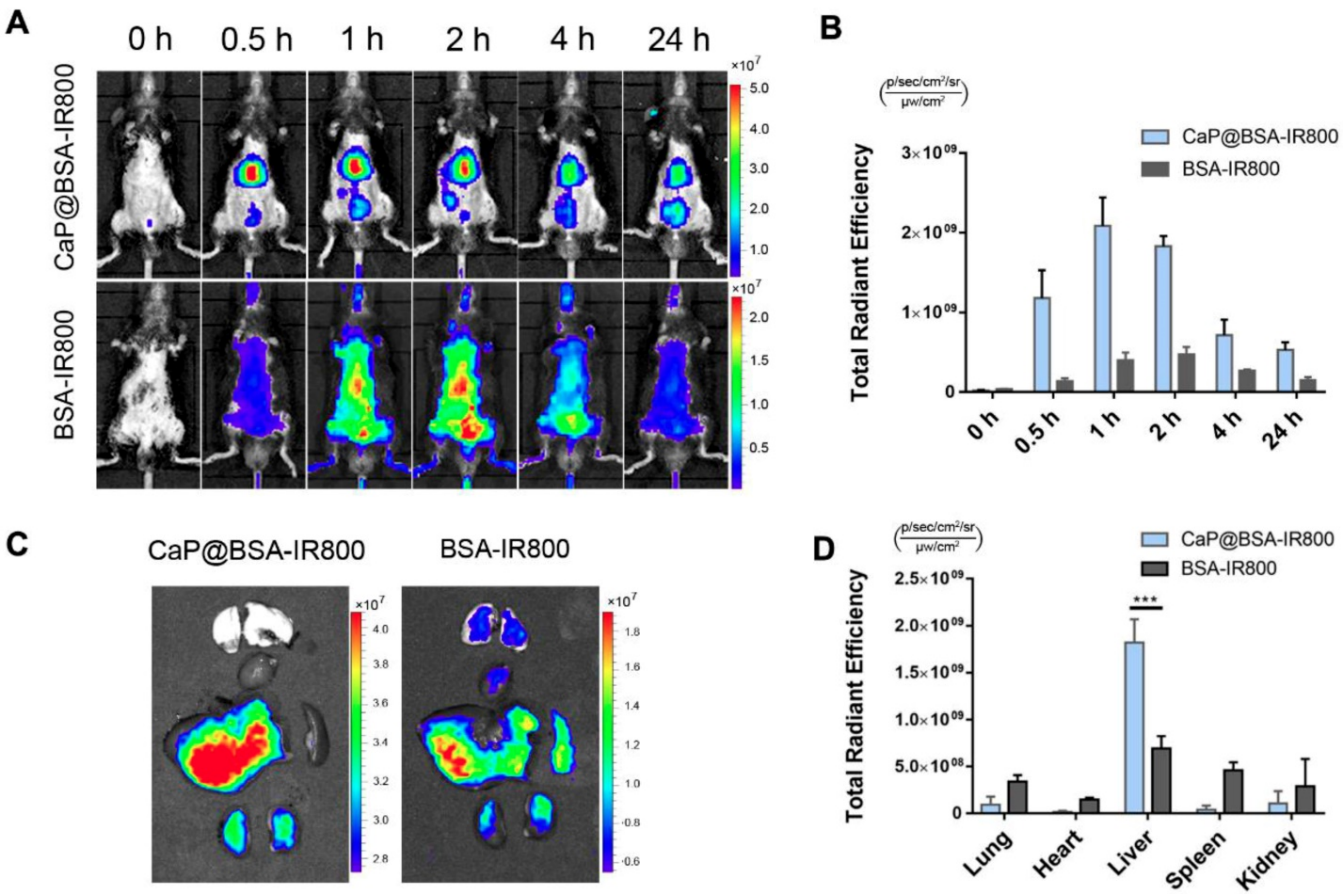
** Liver targeting of CaP@BSA NPs.** A) Representative images of *in vivo* distribution of CaP@BSA-IR800 and BSA-IR800 on fibrotic mice. B) Quantification of the fluorescent intensities of the liver region (n = 3). C) Representative fluorescent images of dissected organs at 2 h after injection of CaP@BSA-IR800 or BSA-IR800. From top to bottom: lungs, heart, liver, spleen, and kidneys. D) Quantification of fluorescence intensities of the five main organs. (n = 3). ***P < 0.001.

To investigate liver targeting of CaP@BSA NPs, a NIR dye IRdye 800CW NHS ester (IR800) was applied to label BSA, and the conjugation was characterized by UV-Vis-NIR and fluorescence spectra (Fig. S18). Then, the IR800 labeled BSA was mineralized to obtain CaP@BSA-IR800 NPs. After intravenous injection of BSA-IR800 or CaP@BSA-IR800 NPs, *in vivo* fluorescence imaging tracking showed significant liver accumulation of CaP@BSA-IR800, while BSA-IR800 exhibited nonspecific whole-body distribution (Fig. 7A). At 0.5 h p.i., CaP@BSA-IR800 had shown 8.6 times higher liver accumulation than BSA-IR800 (Fig. 7B). *Ex vivo* fluorescence images at 2 h p.i. also demonstrated high liver accumulation. To our surprise, the spleen, which also belongs to the reticuloendothelial system and usually exhibits high uptake of ordinary NPs, showed minimal uptake of CaP@BSA-IR800 (Fig. 7C, D).

**Figure 8:**
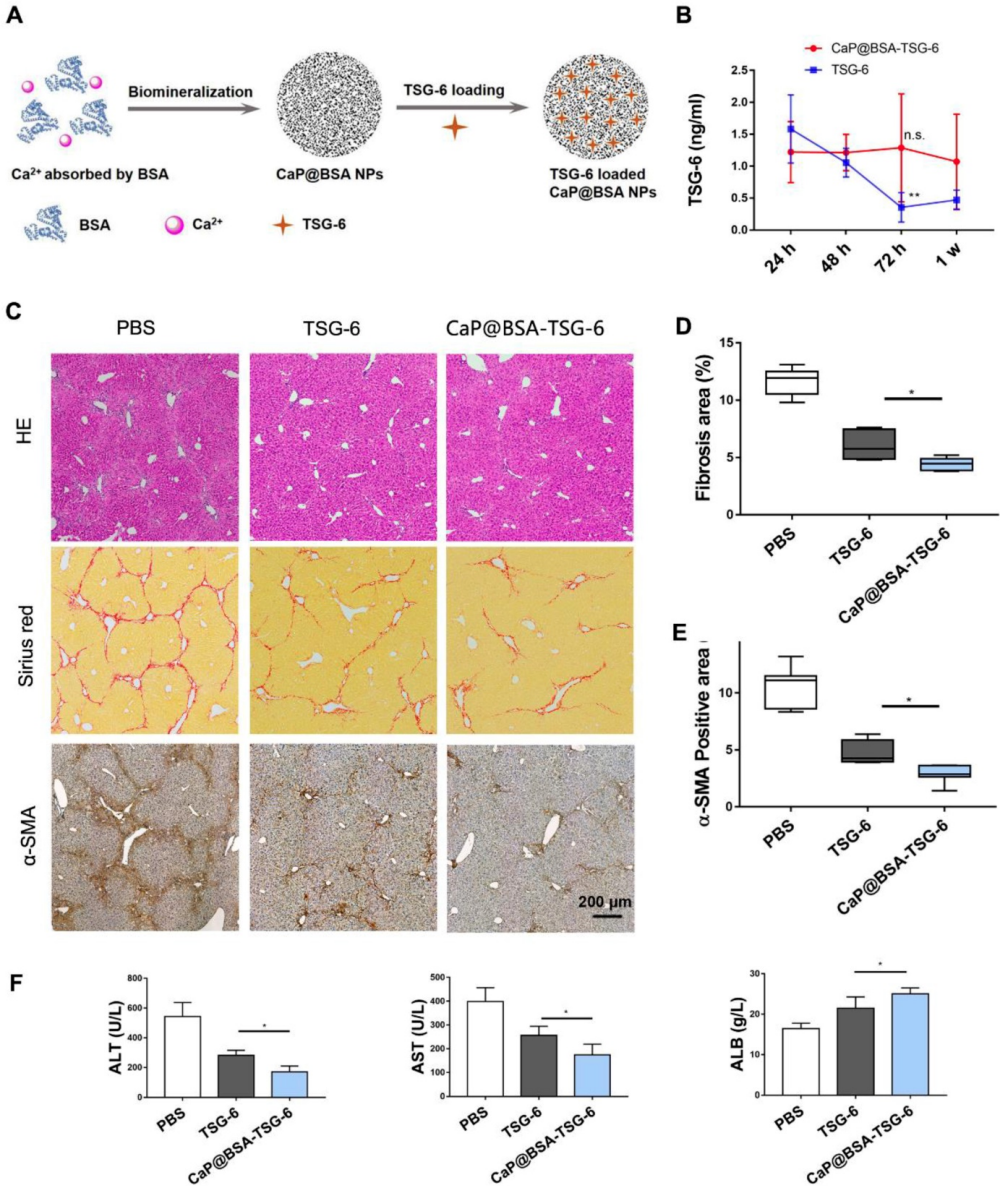
** CaP@BSA-TSG-6 showed improved therapeutic efficacy.** A) The schematic diagram for the preparation of TSG-6 loaded CaP@BSA NPs. B)* In vivo* controlled releasing of TSG-6. 5 *μ*g of CaP@BSA-TSG-6 or TSG-6 was injected intravenously, and serum TSG-6 was tested by ELISA at 24 h, 48 h, 72 h and one week after injection (24 h vs. 72 h). C-F) Therapeutic comparison between CaP@BSA-TSG-6 and TSG-6 on fibrotic mice model. F, histological evaluation; G, percentages of fibrosis area (Sirius red); H, percentages of immunoreactive area (α-SMA). I, biochemical evaluation. n = 6; scale bars, 200 *μ*m; n.s.: non-significance, *P < 0.05, **P < 0.01.

Next, we loaded CaP@BSA with TSG-6 and evaluated its sustained release. Schematic diagram of CaP@BSA-TSG-6 was shown in Fig. 8A. By the intravenously injected the same amount of TSG-6 or CaP@BSA-TSG-6 (at equivalent 5 *μ*g TSG-6) once, the concentration of exogenous human TSG-6 in mice serum was monitored. Both CaP@BSA-TSG-6 and TSG-6 injection increased serum TSG-6 level after 24 h, but free TSG-6 injection group showed quick clearance over time (Fig. 8B, **P < 0.01, 24 h *vs.* 72 h). CaP@BSA-TSG-6 administration, however, resulted in a relatively stable concentration of TSG-6. The TSG-6 level at 72 h was comparable with that of 24 h, which indicated a sustained release of CaP@BSA (Fig. 8B, *P* > 0.05).

The therapeutic effects of CaP@BSA-TSG-6 and free TSG-6 were further compared on the fibrotic mice model. Four weeks later, both CaP@BSA-TSG-6 and TSG-6 injection showed to alleviate fibrosis and decreased myofibroblasts infiltration in comparison with the control group, but CaP@BSA-TSG-6 showed less collagen deposition and α-SMA^+^ areas than that of free TSG-6 treatment (P < 0.05; P < 0.05, Fig. 8C-E). Similarly, CaP@BSA-TSG-6 treatment showed higher ALB levels and less ALT, AST levels than free TSG-6 (Fig. 8F). These results suggested CaP@BSA is an ideal drug nanocarrier for the liver targeting delivery. Moreover, we also prepared CaP@BSA-MMP12 and evaluated its antifibrotic activity. Results showed that CaP@BSA-MMP12 (5 mg) also exhibited therapeutic effectiveness, but a little bit less than the same dose of CaP@BSA-TSG-6 (Fig. S19).

## Discussion

In the present study, we identified TSG-6 as the potent therapeutic cytokine of MSCs in antifibrotic effects and demonstrated TSG-6 promoted M2 polarization and increased MMP12 expression. Based on these findings, we designed CaP@BSA NPs for TSG-6 liver targeting delivery. *In vivo* and *ex vivo* studies showed satisfied therapeutic efficacy of TSG-6 loaded CaP@BSA NPs, and suggested its translational values in the management of liver diseases.

MSCs transplantation is thought to be an effective alternative for liver cirrhosis. However, the mechanisms underlying are not fully understood. Cytokines released from MSCs are crucial for the therapeutic effects. Recent work by An* et al.* showed that injection of the secretome from MSCs could protect against liver fibrosis. In their work, they identified MFGE8 (Milk Fat Globule-EGF Factor 8) playing a critical anti-fibrotic role for MSCs 14. In our study, we selected the 10 most reported cytokines as candidates for evaluation, including MFGE8. However, in our study, changes of MFGE8 were not significant when compared with the changes of TSG-6. This difference may attribute to the complex background of the patient individual and different fibrosis mice model used. By reviewing prior works, TSG-6 aroused our interest because TSG-6 showed strong immunomodulatory effects and tissue-protective properties in many disease models 31-37. Meanwhile, we showed exogenous TSG-6 alone could replicate the therapeutic effects of MSCs, and TSG-6 knockdown hampered MSCs anti-fibrotic effects and failed them in liver fibrosis reversing. These published clues and findings led to the identification of TSG-6 as a potential candidate for our anti-fibrosis research.

MMP12, also called macrophage elastase, is a member of metalloproteinase. MMP12 was found to regulate elastin degradation, which was linked to the maturity of liver fibrosis 27. However, our knowledge of MMP12 is yet to be fully explored. Its protective roles may not confine to the degradation of the extracellular matrix. In this work, we disclosed new protective roles of MMP12 against fibrosis. MMP12 not only suppressed activation of HSCs, which lead to decreased collagen and elastin deposition but importantly, inhibited pro-inflammatory cytokines, such as TNF-α, IL-6, IL-1β. Such importance role of MMP12 was also suggested by a recent study conducted by Kopec *et al.* 38. In their work, Mmp12-deficient mice displayed exacerbated the acetaminophen-induced liver injury, and administration of recombinant MMP12 protein could restore hepatocyte proliferation. Meanwhile, an inhibitory regulation loop between MMP12 and inflammatory cytokines was also identified which provides a new insight of MMP12 antifibrotic role and improves our understanding of antifibrotic mechanisms of MSCs and TSG-6 as well as the knowledge for fibrosis development.

Here, we would like to explain the seemingly “contradictory” results of Fig. 3 and Fig. 4. In Fig. 3D-E, TSG-6 treatment decreased MMP12 levels in the liver, while in Fig. 4D, TSG-6 showed to increase MMP12. In fact, this discrepancy was not a contradiction. Fig. 3 and 4 were describing MMP12 expression at different time point. Fig. 3 showed* in vivo* MMP12 level at two weeks after TSG-6 administration, while Fig. 4 showed *in vitro* MMP12 changes after 24 h TSG-6 induction. There are two possible reasons to may explain the “discrepancy”. Firstly, the exogenous TSG-6 had been gradually consumed *in vivo* after two weeks. Therefore, the induction effects on MMP12 became weak. Secondly, the severity of fibrosis and tissue inflammation was alleviated after two weeks of treatment. The number of anti-inflammatory M2 macrophages would decrease, and since M2 macrophage is the sole source of MMP12 27, it finally led to a decrease of MMP12. Fig. 3F actually depicted similar changes of TSG-6 and MMP12 *in vivo*: The MMP12 was firstly induced by increased TSG-6 due to MSCs infusion, and then to decline gradually because of the TSG-6 consumption caused by MSCs dying. After two weeks, both *in vivo* TSG-6 and MMP12 levels became low.

Currently, there are two distinct approaches to staging of fibrosis. One is a biological approach based on the dosage of serum biomarkers, such as Fibrotest^®^, APRI. Another is physical approach based on ultrasound or imaging techniques, such as transient elastography (TE), magnetic resonance (MR) or single-photon emission computed tomography (SPECT) imaging 39. In our study, we evaluated the potential of MMP12 as fibrosis marker and its relationship with TE. Though MMP12 expression was found to be significantly increased in the cirrhotic patients and F4 cirrhosis stage compared with F1-3 stages of fibrosis, serum MMP12 showed a rather weak correlation with TE score (Fig. S20, r = 0.3302). Thus, MMP12 alone was not reliable for staging fibrosis. However, its potential staging values in combination with other tested fibrotic biomarkers still need to be investigated.

At present, most clinical used anti-fibrotic agents are neither liver nor fibrosis specific. Nanotechnology provides an opportunity to change this scenario 40-44. Tang* et al.* designed a hydrogelator precursor “1-Dex-P” and made a tandem enzymatic strategy of self-assembly to slow release of Dex The 1-Dex-P exhibited much stronger anti-hepatic fibrosis role than free Dex both *in vitro* and *in vivo*^40^. Virender Kumar *et al.* developed an antifibrotic agent (GDC-0449 or miR-29b1) loading polymeric micelle which showed specifically targeting and inactivation of HSCs, one of the primary culprits in hepatic fibrosis 41, 42. The previous study demonstrated that the NPs encapsulated BSA ranged in 100 and 200 nm had good liver targeting ability 30. Similarly, our prepared CaP@BSA NPs with an average size of 98 ± 24 nm and negatively charged exhibited good liver targeting. The IR800 labeled CaP@BSA showed significant accumulation in the liver, while free IR800-BSA exhibited nonspecific whole-body distribution. Furthermore, the Ca^2+^ and PO_4_^3-^ ions released during degradation are completely absorbable and biocompatible and have been proven to have no side effects on various organs (Fig. S16), which is another merit of CaP@BSA. As the reported half-life of TSG-6 was only around 0.15~0.47 h 45, TSG-6 had to be administrated frequently in order to remain in the therapeutic range 34, 46. The CaP@BSA, however, extended TSG-6 releasing period, making TSG-6 therapy much more practical for clinical application as avoiding frequent and unpleasant dosing.

## Conclusions

In conclusion, TSG-6 is a major antifibrotic cytokine of MSCs. TSG-6 exercised antifibrosis by promoting M2 macrophages transition and MMP12 expression. MMP12 could suppress HSCs activation and pro-inflammatory cytokines release. Importantly, we found an inhibitory loop between MMP12 and pro-inflammatory cytokines and discovered an MMP12 rescue effect of TSG-6, which may partly explain the aggravating process of cirrhosis and antifibrotic mechanisms of TSG-6 and MSCs. TSG-6 loaded CaP@BSA NPs exhibited specific liver targeting and improved therapeutic effects, which indicated potential translation values for the management of liver diseases.

## Supplementary Material

Supplementary materials and methods, figures, and tables.
