# Supplementary material for: Liver-targeted delivery of TSG-6 by calcium phosphate nanoparticles for the management of liver fibrosis
Source: Theranostics. 2020 Jan 1;10(1):36–49. doi: 10.7150/thno.37301 (PMC6929629; doi:10.7150/thno.37301)
Supplement: Supplementary file 1 — Supplementary materials and methods, figures, and tables. [file thnov10p0036s1.pdf]

Supplementary materials and methods

1. Cell culture

Human umbilical cord-derived mesenchymal stem cells (MSCs) were obtained from the National Engineering Research Center (Tianjin AmCellGene Engineering Co., Ltd, China). MSCs isolated from the umbilical cord of natural birthed full-term fetus were passaged in mesenchymal stem cell expansion medium (CCM004, R&D Systems, MN) with 5% CO_2 and saturated humidity at 37 ℃. The characterization of MSCs was determined by flow cytometry. The antibody conjugated with PE- or FITC (anti-CD29, CD90, CD105, CD73, CD44, CD45, CD34, and HLA-DR) were purchased from BioLegend (San Diego, CA). Analyses were performed using FACScan (BD Bioscience, CA). Osteoblast, adipocyte, and chondrogenic differentiation mediums were purchased from Cyagen Biosciences (HUXUC-90021, HUXUC-90031, HUXUC-9004, Guangzhou, China) and initiated according to the manufacturer's instructions. Intracellular lipid or Calcium deposits can be stained with Oil Red O or Alizarin Red S method. Proteoglycans presence which indicated chondrogenic differentiation was verified by toluidine blue after 21 d of pellet induction in a 15 mL tube.

Human hepatic stellate cells (LX2) was purchased from Procell Life Science & Technology Co. Ltd. (Wuhan, China). LX2 cells were resuscitated in Dulbecco's Modified Eagle's medium (DMEM) (Sigma-Aldrich, MO) supplemented with 10% fetal bovine serum (FBS, Gibco, NY) and maintained in DMEM supplemented with 1% FBS. Cells were incubated at 37 ℃ in a humidified atmosphere supplied with 5% CO_2. For TGF-β1 (Merck Millipore, MA) stimulations, LX2 cells were activated with 2 ng/mL TGF-β1 in DMEM supplemented with 1% FBS for 24 h. For MMP12 intervention, cells were pre-treated with different concentrations MMP12 (917-MPB, R&D Systems, MN, 1, 10, 100, 250 ng/mL) for 30 min before the addition of TGF-β1.

2. NIR dye labeling and *in vivo* distribution

Near-infrared dye, 1,1'-dioctadecyl-3,3,3,3'-tetramethylindotricarbocyanine iodide (DiR; DiIC18(7), Molecular Probes, Invitrogen, CA) was applied for labeling MSCs cells as we previously reported [1]. The DiR working solution was prepared in 10 mL PBS containing 3.5 μg/mL dye and 0.5% ethanol. The labeling was done by incubation MSCs with DiR dye for 30 min at 37 °C. Cells were then washed and centrifuged twice by the fresh medium at 1,500 rpm to ensure complete removal of any

unbound dye.

Fibrosis mice receiving DiR-MSCs were sacrificed at day 1, 3 and 7 after transplantation. The peritoneal cavity was exposed and organs (heart, liver, spleen, lungs, bowel, and kidneys) were extracted for organ distribution. All imaging procedures were conducted using small animal imaging system (IVIS Kinetics, Caliper Life Science). Signal intensity was quantified and processed using the Living Image software (V5.0, Caliper Life Science). GFP^+ human umbilical cord-derived MSCs was purchased from Cyagen Biosciences (HUXUC-01101, Guangzhou, China). GFP^+ MSCs transplantation was done with the same procedure mentioned above. The engraftment of GFP^+ Cells in the liver was evaluated by frozen section and counted by Image-Pro Plus software (v6.0, Media Cybernetics Inc.).

3. TSG-6 knockdown

The lentivirus (Lv-shTSG-6, Lv-nc) were packaged by GeneChem (Shanghai, China). MSCs transfection procedures were conducted according to the manufacturer's instructions. The MSCs transfected with Lv-shTSG-6, Lv-nc were named as Lv-shTSG-6 MSCs, Lv-nc MSCs respectively. Knockdown efficiency was assessed by qRT-PCR, ELISA and western blot.

4. MSCs stimulation

The stimulation medium was prepared by DMEM containing 20% (v/v) pooled sera. The serum samples were taken from liver cirrhotic patients (n = 5; hepatitis B without active viral replication) and healthy volunteers (n = 5). Study protocols were reviewed by the Ethics Committee of Xijing Hospital and informed consents were obtained from each patient and volunteer. The clinical information was listed in Supplemental Table S1. MSCs were seeded in 6-well plates with a density of 3×10^5 cells/well and treated with above stimulation medium for 24 h. Then the supernatant and cells were collected for further analysis.

5. Bone marrow-derived macrophages isolation and phenotype induction

Primary macrophages were extracted from the bone marrow of C57BL/6J mice as reported [2]. The C57BL/6J mouse (4–6 week) was sacrificed and immersed in 75% ethanol. Then the legs and tissues were removed. Femur cavities were flushed using a 27 g needle syringe with Dulbecco's phosphate-buffered saline (DPBS). The cells were seeded at a density of 2×10^6 cells/mL and incubated

in DMEM supplemented with 10% FBS, 1% streptomycin/penicillin plus 20 ng/mL macrophage colony-stimulating factor (M-CSF, 315-02, PeproTech, NJ) for 7 days. Characteristics of macrophages were evaluated by flow cytometry analysis and immunofluorescence (IF) staining of F4/80 and CD11b expression. For phenotype induction, macrophages were plated on the lower compartment of 6-well Transwell® at a density of 3×10^5 cells/well and cultured with DMEM with 10% FBS. Classic M1 phenotype was induced by 100 ng/mL of LPS (L2640, Sigma, MO) and 20 ng/mL of IFN-γ (315-05, PeproTech, NJ). The M2 phenotype was induced by 20 ng/mL of IL-4 (214-14, PeproTech, NJ). To analyze the effects of TSG-6 and MSCs effects on macrophage phenotype switch, 1.5×10^5 MSCs or LV-shTSG-6 MSCs or 100 ng/mL TSG-6 were seeded or added into the upper compartment of Transwell® and co-cultured with macrophages for 24 h. To explore the effect of MMP12 on M1 polarization, macrophages were pre-treated with MMP12 (250 ng/mL) for 30 min before the M1 induction. To explore the effect of pro-inflammatory cytokines (such as TNF-α, IL-6 and IL-1β) or TSG-6 on MMP12 expression, macrophages were pre-treated with TNF-α (40 ng/mL, 315-01A, PeproTech, NJ), IL-6 (40 ng/mL, 216-16, PeproTech, NJ), IL-1β (40 ng/mL, AF211-11B, PeproTech, NJ), Mix (TNF-α, IL-6 and IL-1β), TSG-6 (200 ng/mL) or TSG-6+Mix for 30 min prior to initiating M2 induction.

6. Primary liver macrophages isolation and supernatant collection

Primary liver macrophages were isolated using a modified two-step collagenase perfusion method [3]. Briefly, mice were anesthetized, and the peritoneal cavity was exposed and a cannula was inserted into inferior vena cava. Then the mice liver was flushed with Ca^{2+}-free perfusion buffer at 37 ℃ to remove the blood at a flow rate of 20 mL/min for approximately 10 min. The entire liver was excised and the connective tissues were carefully removed. Then the liver was digested with 30 mL of collagenase IV buffer (17104019, Gibco, NY) at 37 ℃ with a flow rate of 10 mL/min for 15 min. The catheter was then removed and the liver was transferred to a dish containing 20-30 mL DMEM. The Glisson's capsule of the liver was peeled back and the cells were shacked out in the liquid. The cell suspension was filtered through a 100 μm gauze mesh filter. Hepatocytes were separated from nonparenchymal cells by three rounds of low-speed centrifugation at 50 g. The supernatant containing the macrophages cells was collected. Slowly layer the supernatant on the Percoll gradient with a 15 mL

pipette and centrifuge the mixture at 2,300 rpm for 30 min at 4 ℃. Carefully collected the middle interphase and transfer the cells to a new 50 mL tube. Then the cell precipitates were collected after centrifugation at 1,500 rpm for 10 min at 4 ℃. To determine the effects of different treatment (PBS, MSCs, Lv-shTSG-6 MSCs, TSG-6) on cytokines releasing, the primary liver macrophages were isolated at 24 h post cell transplantation. Cultured *in vitro* for another 24 h, the supernatant of culture medium was collected for ELISA and macrophages were harvested for PCR analysis.

7. Histological evaluation

The tissues were fixed in 4% paraformaldehyde, dehydrated in graded ethanol, cleared in xylene, and embedded in paraffin. The paraffin-embedded sections (5 μm) were stained with hematoxylin and eosin (HE), for routine histological examination and Sirius Red for fibrosis evaluation. Quantification of collagen fiber was assessed using Image-Pro Plus software (v6.0, Media Cybernetics Inc.).

8. Measurement of blood biochemistry

The mice serum obtained from each time point, and the level of alanine aminotransferase (ALT), aspartate aminotransferase (AST) and albumin (ALB) were determined using an automatic biochemistry analyzer (Hitachi 7600-120, Hitachi, Japan) [4].

9. Immunofluorescence (IF) staining

Tissue sections were prepared and incubation with anti-α-SMA antibody (A2547, 1:500, Sigma, MO), anti-TSG-6 antibody (MAB2104, 1:200, R&D Systems, MN), anti-F4/80 antibody (AB6640, 1:100; Abcam, MA), anti-iNOS antibody (AB15323, 1:100, Abcam, MA) or anti-CD206 (AB64693, 1:100; Abcam, MA) overnight at 4 °C. After washing triple times with PBS, the Alex fluor 488, 594-conjugated secondary antibody (Jackson ImmunoResearch, PA) for 1 h at room temperature. After counterstaining with DAPI (Roche, Basel), the sections were mounted using anti-fade solution. Percentages of positive cells were analyzed by Image-Pro Plus software (v6.0, Media Cybernetics Inc, Bethesda, MD).

10. RNA extraction and quantitative real-time PCR (qRT-PCR)

Total RNA was extracted by RNAeasy Plus kit (74134, QIAGEN, Duesseldorf) and reverse transcription was done by PrimeScript™ RT Master Mix (RR036A, Takara, Tokyo). TB Green Premix

Ex Taq II (DRR820A, Takara, Tokyo) and CFX96 Touch™ real-time PCR System (Bio-Rad, CA) were used for amplification. Specific primer for each transcript was list in Supplemental Table S6. All reactions were performed in triplicated. GAPDH mRNA was used as an internal control to normalize mRNA expression.

11. Immunoblotting

The proteins from cells or tissue samples were prepared by the RIPA lysis buffer (Beyotime Biotechnology, China) with proteinase inhibitors and phosphatase inhibitors (Roche, Basel). Extracted protein lysates were quantified by Bradford method using Bio-Rad protein assay (Bio-Rad Laboratories, CA) and 12 μg of protein was loaded to SDS-PAGE, followed by transfer to nitrocellulose membranes (Bio-Rad Laboratories). Membranes were blocked with TBS-T containing 5% skim milk. After blocking, the membrane was incubated with primary antibodies overnight at 4 ℃. Information of used primary antibodies was listed in Supplementary Table S7. After 30 min incubation with peroxidase-conjugated secondary antibodies (Jackson immune research laboratories, PA), the blots were developed by an enhanced chemiluminescence kit (Thermo Fisher Scientific, IL). Image Lab software was used to quantify the western blot bands.

12. ELISA

The human TSG-6 and MMP12 levels were measured using ELISA kit (ELH-TSG6, ELH-MMP12, RayBiotech, GA). The levels of MMP12 in patients with hepatitis B virus-related cirrhosis and primary biliary cirrhosis were detected respectively. The clinical information of patients in these two groups was shown in supplemental Table 4 and Table 5 respectively. The mice MMP12 changes were determined by ELISA kit (AB213878, Abcam, MA). The levels of immunomodulatory factors (IL-6, TNF-α, IFN-γ, IL-12p70, IL-4, IL-10) in primary liver macrophages were assessed by ELISA (M600B, MTA00B, MIF00, M1270, M400B, M1000B, R&D Systems, MN). All assays were performed according to the manufacturers' protocols.

13. Microarray assays of hepatic mRNA

Total RNA isolated from livers of fibrosis mice with or without TSG-6 treatment was studied by mouse gene expression microarray (n = 3, SurePrint G3 Gene Expression Microarrays, Agilent

Technologies, Inc, CA). The experimental procedure was performed in accordance with manufacturer's instructions, and data interpretation was done by CapitalBio (CapitalBio Technology Co. Ltd, China). The GEO ID of the expression profile data is GSE121669.

14. IHC

Human tissue array containing 28 cases of liver fibrosis and 16 cases of normal liver were brought from Alenabio (LV20812a, Xi'an, China) and standard procedures of immunohistochemistry staining (IHC) were strictly followed. Briefly, sections were dewaxed and rehydrated by xylol and alcohol. After antigen retrieval, 1% H_2O_2 was introduced to block endogenous peroxidase activity. After 30 min goat serum for blocking, the slices were incubated either with anti-MMP12 antibody (Abcam, ab52897) overnight at 4 °C. Information of used antibodies was listed in Supplemental Table S7. After washing with PBS, the sections were incubated 1 h at room temperature with secondary antibody, then visualized by diaminobenzidine (DAB).

15. Characterization of CaP@BSA NPs

Fourier-transform infrared (FT-IR) spectra were obtained on Spectrum Two (PerkinElmer, MA) with the wavenumber range from 4,000 to 400 cm^{-1} at 4 cm^{-1} resolution and 32 scans per sample. The morphologies of CaP@BSA nanoparticles (NPs) were observed by field-emission scanning electron microscopy (FE-SEM, Hitachi SU-70, Japan, accelerating voltage 5 kV) and transmission electron microscopy (TEM, JEM 1230, Japanese Electronics & America GATAN, accelerating voltage 80 kV). The size distributions of CaP@BSA nanospheres were obtained from the TEM image using Image J software (NIH, MD). Elemental mapping based on energy-dispersive X-ray spectroscopy (EDX) was recorded on an EI Magellan 400 with an accelerating voltage of 25 kV. Thermogravimetric (TG) analysis was measured on an STA 449C simultaneous thermal analyzer (Netzsch, Germany) with a heating rate of 10 ℃/min in the flowing air from room temperature to 800 ℃. The surface charge of the NPs was obtained by a zeta-potential analyzer (ZetaPlus, Brookhaven Instruments Corporation, NY). The contents of Ca elements were measured using an inductively coupled plasma instrument (JY 2000–2, Horiba, France).

16. Preparation of CaP@BSA-IR800

Firstly, BSA was labeled by IRdye 800CW NHS ester (IR800) according to the manufactory instructions[5]. Briefly, 50 mg BSA was dissolved in 5 mL saline, the pH value was adjusted to 8.5–8.7 by using Na_2CO_3 aqueous solution (50 mM). Then, a certain volume of IR800 (10 mM) in DMSO was added in the above solution under vigorous stirring, the molar ratio of dye to BSA was set as 1.8, and the DMSO concentration was kept below 2% (v/v) in the reaction system. After stirring for 2 h under room temperature, the reaction mixture was purified by centrifugal ultrafiltration (10k MWCO, 8,500 rpm, 5 min), and concentrated by DMEM to 1 mL. The UV−Vis absorption and fluorescence spectra of the IR800 labeled BSA (IR800-BSA) were detected on a Carry 60 UV-Vis spectrophotometer (Agilent Technologies, CA and a fluorescence spectrophotometer (Thermo Scientific Lumina, Germany), respectively. For the preparation of CaP@BSA-IR800, 200 μL of BSA-IR800 was added in 0.8 mL DMEM medium, sealed, and incubated at 37 ℃ for 24 h to reach equilibrium. Then, 10 μL $CaCl_2$ (1 M) was added into the reaction system for another 24 h. After incubation, the product was separated by centrifugation (13,500 rpm, 15 min), washed by deionized water for several times.

17. *In vitro* TSG-6 loading and releasing

The typical *in vitro* drug loading and releasing experiments were carried out as following: CaP@BSA NPs (240 μg) were dispersed in 240 μL PBS 7.4 containing 30 μg TSG-6. The suspension was sealed and then shaken at a constant rate (120 rpm) at 37 ℃ for 4 h. The drug-loaded product was separated by centrifugation, and the remained TSG-6 in the supernatant was determined by the TSG-6 ELISA kit (R&D Systems Inc.) according to the standard protocol. The drug loading efficiency was calculated by the formula: drug loading efficiency = ((original TSG-6 - remained TSG-6)/original TSG-6) × 100%. For the release experiments, the obtained TSG-6 loaded CaP@BSA NPs was re-dispersed in 720 μL PBS 7.4, and then sub-packed in three tubes (240 μL per tube) at 37 ℃ with constant shaking (120 rpm). The drug release medium (120 μL) was withdrawn for analysis at given time intervals and replaced with the same volume of fresh PBS. At each time point, the reaction system was centrifugated to separate released drug and solid particles. The released drug was determined by the TSG-6 ELISA kit (R&D Systems Inc.) and normalized to the total released drug amount, which was determined by the complete dissolution of particles at pH 5.

18. *In vivo* imaging and drug-releasing

The liver targeting was evaluated by a small animal imaging system (IVIS Kinetics; Caliper Life Science, Hopkinton, MA). BSA-IR800 and CaP@BSA-IR800 NPs were dispersed in PBS 7.4 and then injected to mice (2 mg/kg, 200 μL). The fluorescence images were accomplished at 0.5, 1, 2, 4, and 24 h after injection. Fluorescent intensity was quantified and processed using the Living Image Software (V5.0, Caliper Life Science).

0.2 mg CaP@BSA NPs were dispersed in 0.2 mL PBS 7.4 that containing 25 μg TSG-6. The resulting mixture was sealed and then shaken at a constant rate (120 rpm) at 37 ℃ for 4 h to obtain TSG-6 loaded CaP@BSA. To evaluate *in vivo* drug-releasing efficiency, 40 μL of TSG-6-loaded CaP@BSA suspension (containing *5* μg TSG-6) was mixed with 160 μL PBS and injected to mice. Mice injected with *5* μg free TSG-6 in 200 μL PBS were treated as control. The serum TSG-6 concentrations were dynamically measured by ELISA (Raybiotech, GA) at the indicated time point.

19. The pH-responsive degradation experiments

The degradation experiments of CaP@BSA NPs was carried out in phosphate buffer solution (PBS) with different pH values (pH 7.4, 6.8, 6.5, 6.2, and 5.0) at 37 ℃ under constant shaking (120 rpm). Briefly, 1 mL CaP@BSA suspension (5 mg/mL) was placed in dialysis bags (MWCO = 3500), and then the dialysis bag was sealed to dialysis against another 19 mL of PBS at pH 7.4, 6.8, 6.5, 6.2, and 5.0 respectively to simulate different biological environments. The supernatant solution (5 mL) was withdrawn for ICP analysis to measure the concentrations of Ca^{2+} ions at given time intervals and replaced with the fresh PBS with the same volume and the same pH value.

20. Biocompatibility of BSA-CaP NPs.

The biocompatibility of CaP@BSA NPs was evaluated by 3-(4,5 dimethylthiazol-2-yl)-2,5-diphenyltetrazollium bromide (MTT) assay. The cells were cultured in RPMI-1640 medium supplemented with 10% fetal bovine serum and 1% penicillin-streptomycin at 37 ℃ for 48 h. Then, the cells were seeded in 96-well flat-bottom microassay plates at a concentration of 5×10^3 cells per milliliter and incubated for 24 h. After that, the media were replaced with fresh media containing CaP@BSA NPs (10−400 μg/mL) and co-cultured for 48 h. The sample-free tissue culture plate was used

as the control. Cell viability was quantified by MTT assay and calculated based on the formula: cell viability = (OD490 nm of the sample/OD490 nm of the control) $\times$100%, the cell viability of the control group was set as 100%, and the data represents the mean value of four parallel measurements.

21. Hemolysis assay

The red blood cells (RBCs) were isolated from serum by centrifugation of the mixture containing 0.5 mL blood sample and 1 mL PBS 7.4 at 4,500 rpm for 3.5 min. After washed by PBS for five times, the purified RBCs were diluted to 5 mL. Then, 0.3 mL of diluted RBCs suspension was added to the quadruple volume of PBS with different concentrations of CaP@BSA NPs (25−800 μg/mL). The mixtures were vortexed and kept to stand for 4 h at room temperature. These samples were then centrifuged to measure the absorbance of the supernatants at 541 nm by using an UV-vis spectrophotometer. The RBCs treated with deionized water and PBS were set as positive and negative controls, respectively.

Supplementary figures and tables

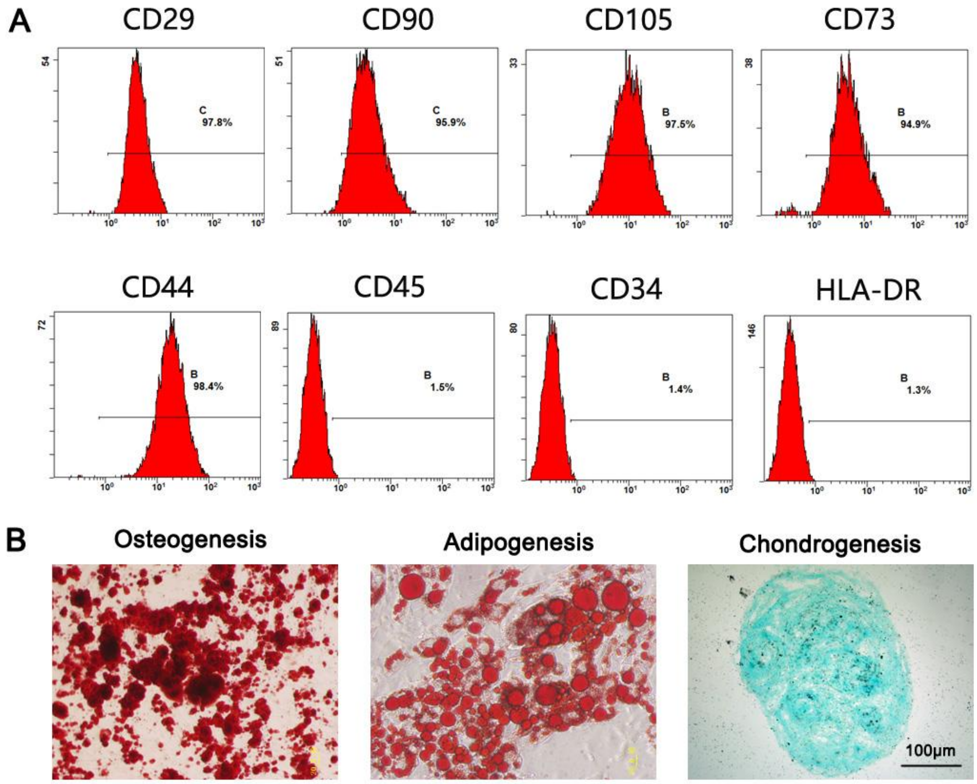

Fig. S1. Characterization of MSCs. (A) Representative flow cytometry plots showing MSCs expressing the surface markers CD29, CD90, CD105, CD73 and CD44 but not CD45, CD34 and HLA-DR. (B) Multipotential capabilities of differentiating into osteoblasts, adipocytes, and chondrocytes. From left to right: alizarin red, oil red O, and alcian blue staining. Scale bar, 100 μm.

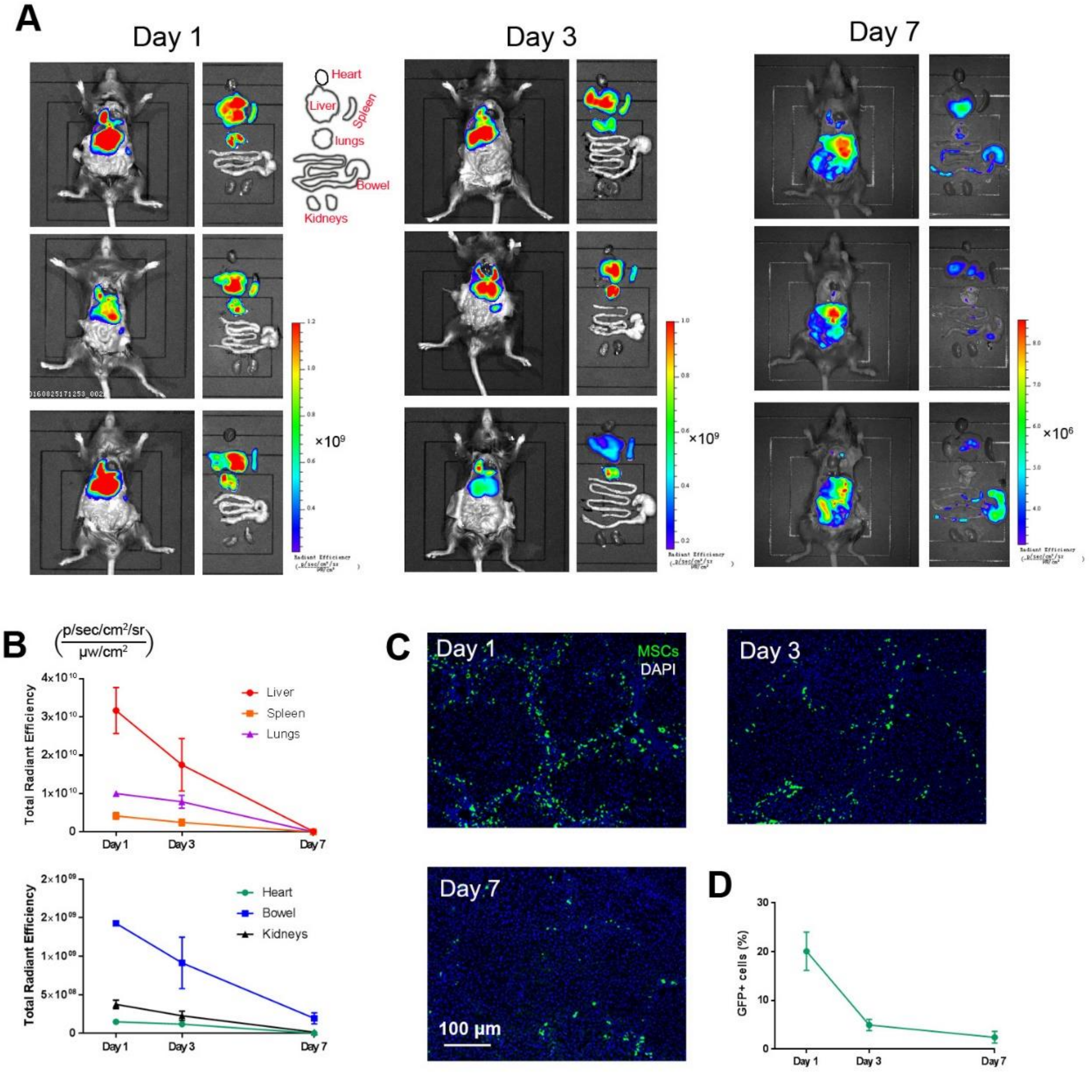

Fig. S2. MSCs distribution in CCL_4 induced fibrotic mice. (A) MSCs labeled with DiR dye were given by tail vein. Imaging was taken at day 1, 3 and 7 post-injection. Left panel: whole-body imaging; Right: organ imaging (From top: heart, liver, spleen, lungs, bowel, and kidneys). (B) MSCs signal quantification of six main organs (liver, spleen, lungs, heart, bowel, and kidneys). (C) Liver sections showing GFP^+ MSCs at day 1, 3 and 7after GFP^+ MSCs transplantation. The MSCs mainly engrafted at the hepatic portal area. (D) Percentage of GFP^+ MSCs in the liver section. Scale bar, 100 μm.

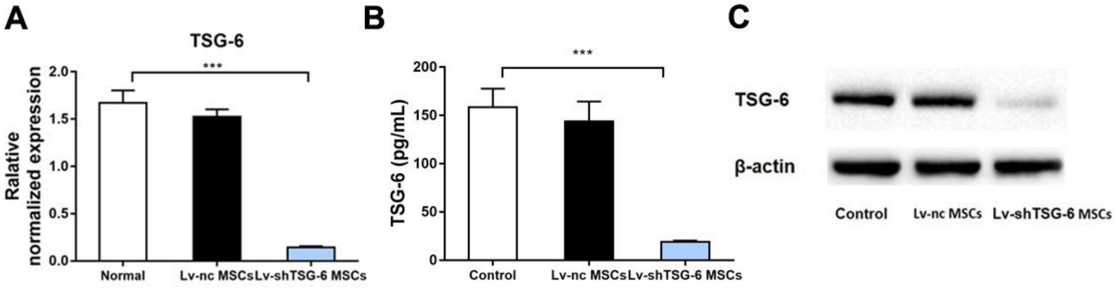

Fig. S3. Establishment of TSG-6 knockdown MSCs. MSCs were transfected with Lv-shTSG-6 and Lv-nc. The knockdown efficiency levels were verified by real time-PCR (A), ELISA (B) and Western blot (C). ***$P < 0.001$.

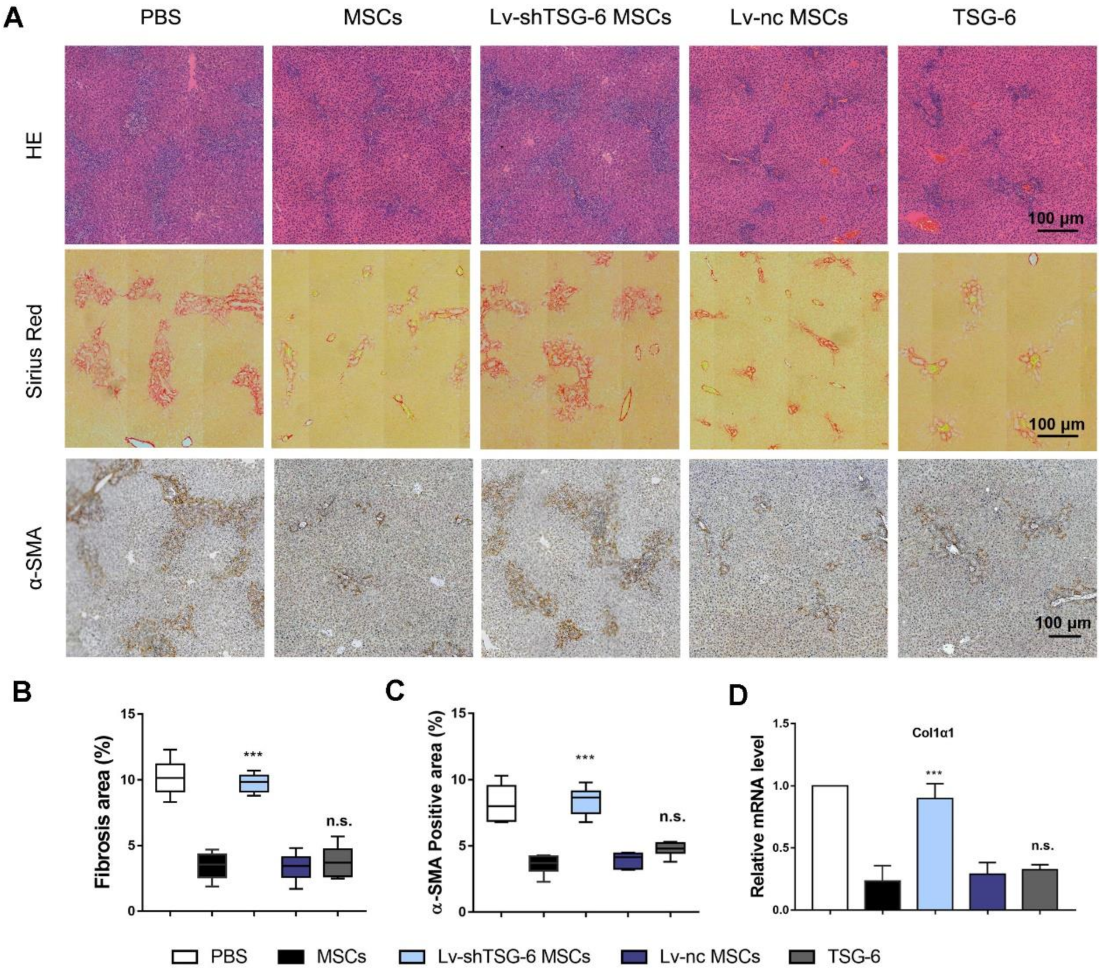

Fig. S4. TSG-6 ameliorated liver fibrosis of bile duct ligation (BDL) cholestatic fibrotic model. BDL model was established as previously reported [2]. PBS, MSCs, Lv-shTSG-6 MSCs, Lv-nc MSCs, and TSG-6 were infused intravenously to BDL mice. The transplanted MSCs number, TSG-6 dosage, and injection frequency were as same as CCL_4 model. The mice were sacrificed 14 days after the cell infusion. (A) Liver sections were stained with HE, Sirius red staining or anti-α-SMA antibody. Scale bar, 100 μm. (B-C) Quantification of fibrotic (Sirius red) and α-SMA^+ areas. Percentages were measured by Image-Pro Plus. (D) Quantitative PCR analysis of collagen1α1, another marker of collagen deposition. n = 6. ***$P < 0.001$; n.s.: non-significance; Compared to MSCs group.

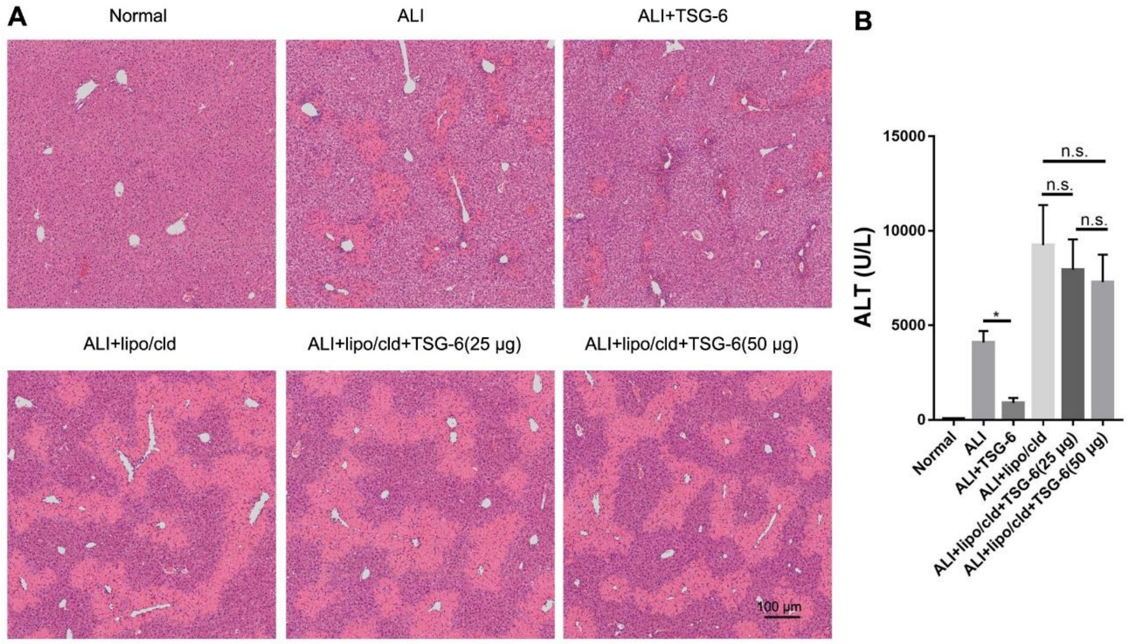

Fig. S5. Macrophages were necessary for TSG-6 antifibrotic function. Acute liver injury was induced by intraperitoneal administration 30 ng/g body weight of LPS. To deplete *in vivo* macrophages, 150 μL clodronate liposomes was intravenously injected 2 days prior to LPS administration [6]. Mice were sacrificed for analysis at 48 h after TSG-6 injection. (A) Representative HE pictures showing necrosis area differences of TSG-6 with or without macrophages presence. Scale bar: 100 μm; (B) serum ALT levels of different indicated groups. n = 5-6, *, $P < 0.05$; n.s., non-significance.

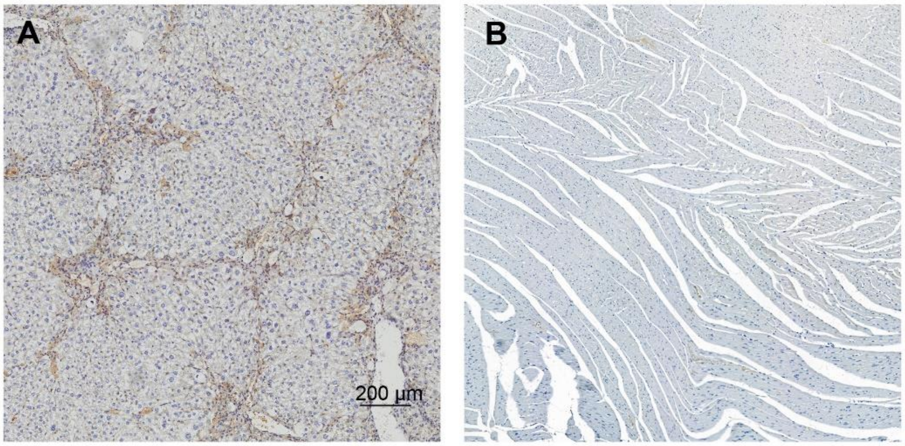

Fig. S6. MMP12 expression in positive and negative control tissue. MMP12 was highly expressed in fibrotic liver tissue as a positive control (A), while it was not expressed in normal heart tissue as a negative control (B).

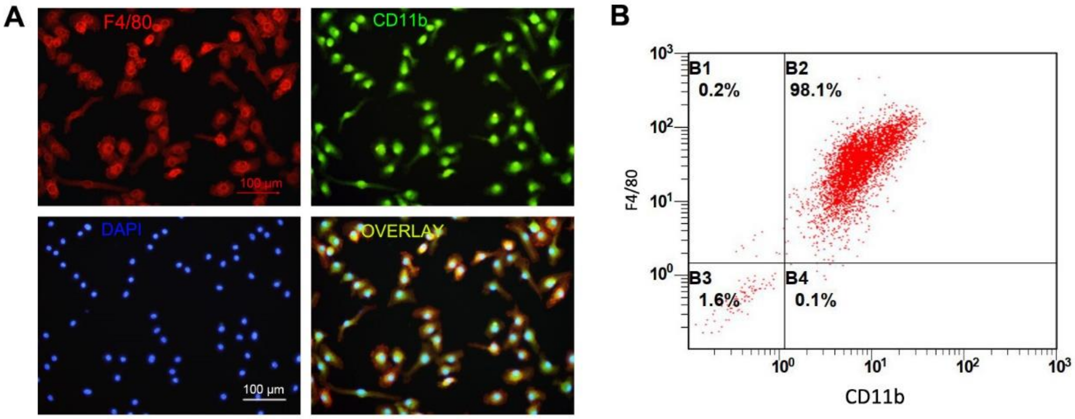

Fig. S7. Isolation macrophages from bone marrow. (A) Macrophages marker were analyzed by IF with Alexa594-labeled anti-F4/80 (red) and FITC-labeled anti-CD11b (green). Cell nuclei were counterstained with DAPI. Scale bar, 100 μm. (B) Cells were examined by FACS after staining with anti-mouse F4/80 and anti-CD11b antibodies. The isolation purity was nearly 95%.

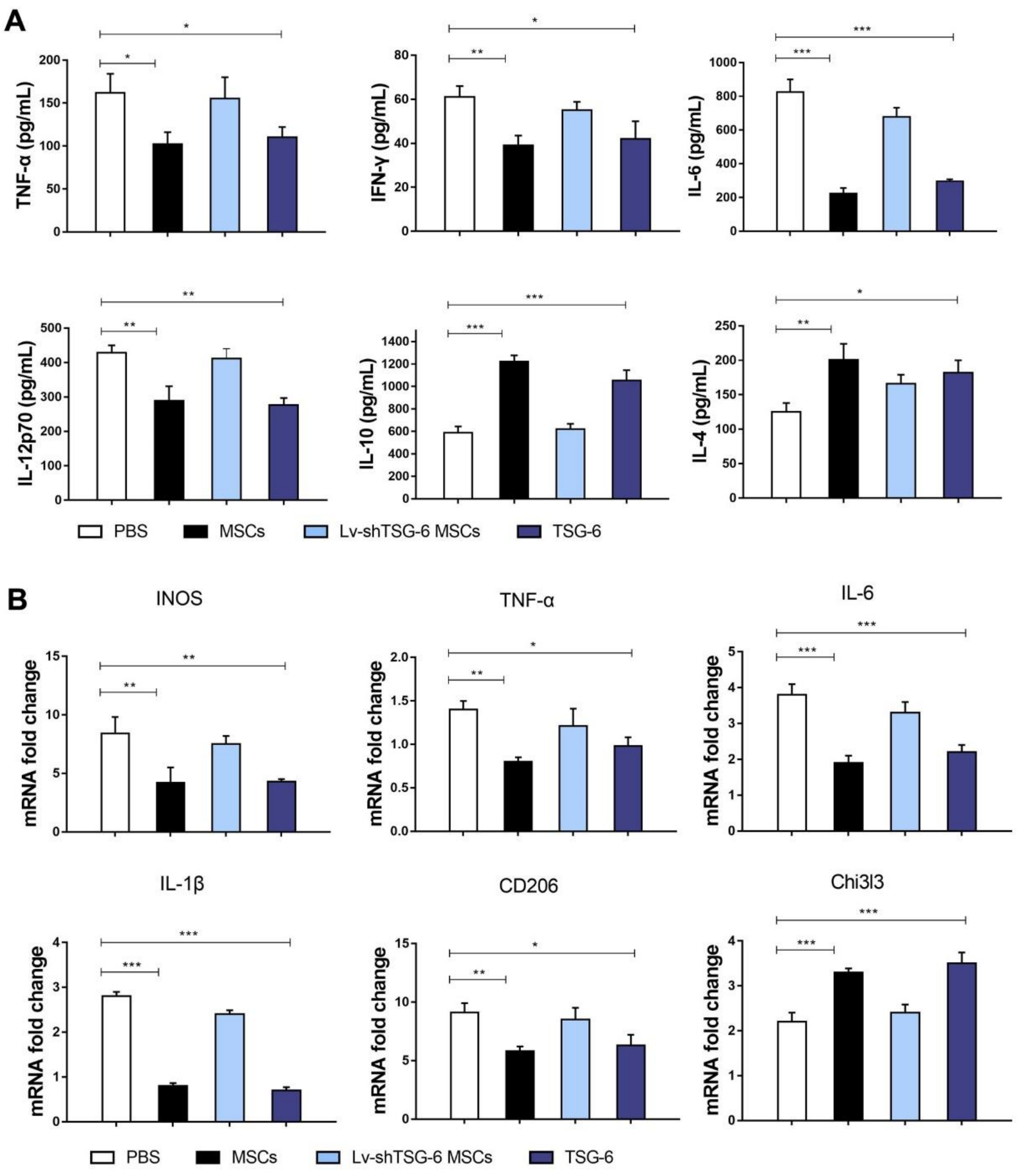

Fig. S8. TSG-6 treatment increased liver macrophages to express anti-inflammatory factors. (A) Levels of pro-inflammatory cytokines (TNF-α, IFN-γ, IL-6, and IL-12p70) and anti-inflammatory cytokines (IL-10 and IL-4) in cultured supernatants of liver macrophages. (B) Quantification of mRNA of iNOS, TNF-α, IL-6, IL-1β, CD206, and Chi3l3 by qRT-PCR. n = 4. *$P < 0.05$, **$P < 0.01$, *** $P < 0.001$.

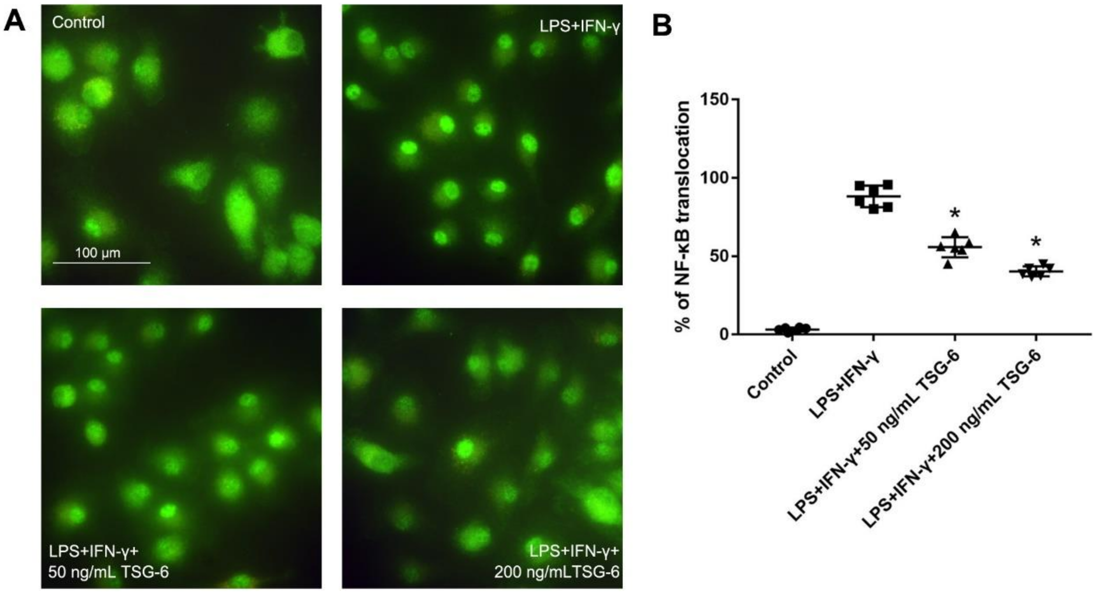

Fig. S9. Effects of TSG-6 on LPS+IFN-γ induced nuclear translocation of NF-κB. (A) Representative IF images showing NF-κB p65 (green) nuclear translocation in primary macrophages stimulated by LPS +IFN-γ with or without TSG-6. (B) Quantitation of NF-κB p65 nuclear translocation in each indicated group. Results are expressed as the percentage of the p65 nuclei-positively stained cells to the total cells. (Scale bar:100 μm, *$P < 0.05$, compared to LPS +IFN-γ treatment)

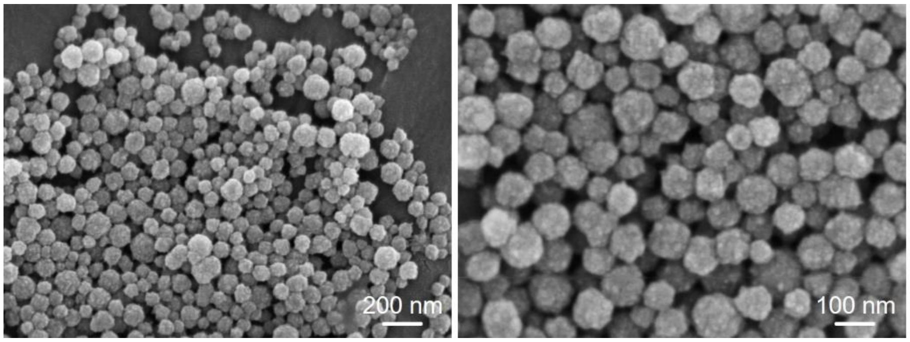

Fig. S10. SEM images of CaP@BSA nanospheres.

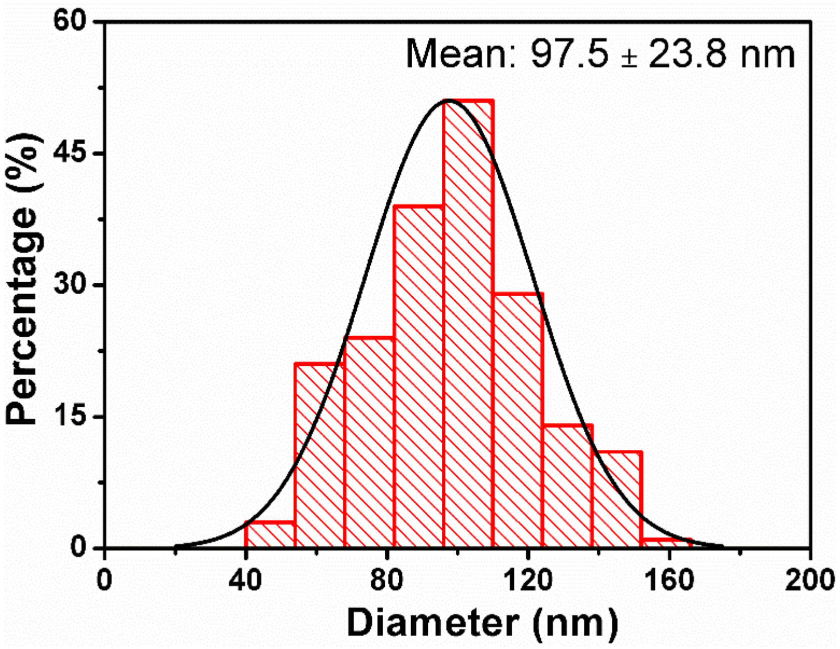

Fig. S11. The size distribution of CaP@BSA nanospheres.

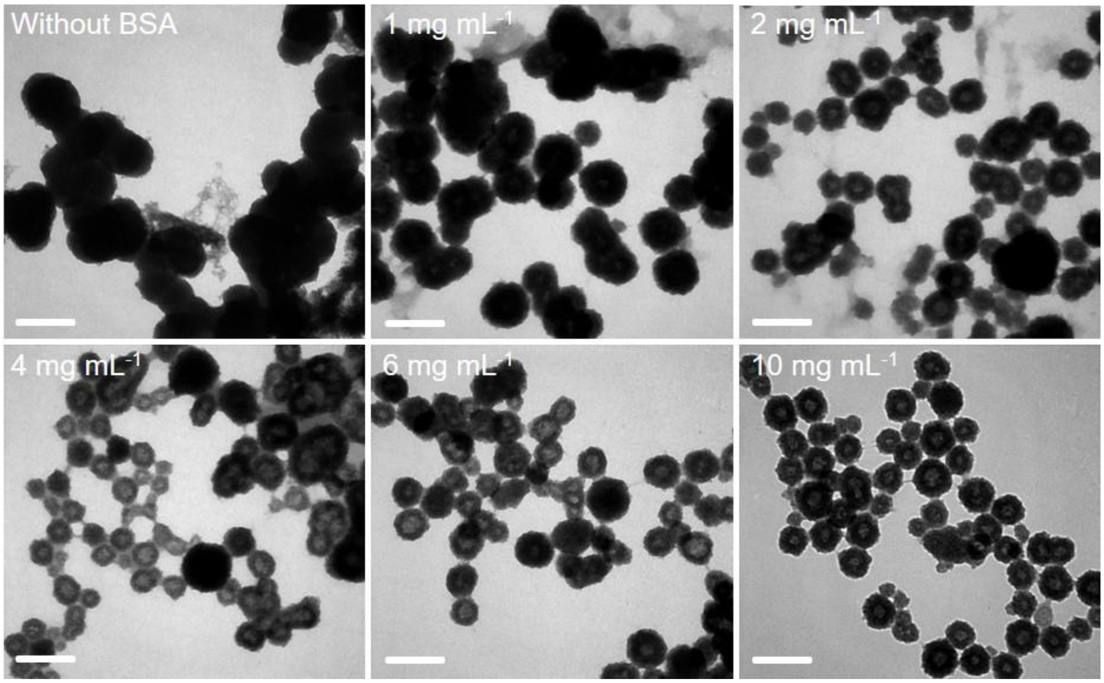

Fig. S12. TEM images of CaP@BSA NPs prepared with different concentration of BSA. Top left corner: Negative control, mineral particles formed without BSA. Scale bar: 200 nm.

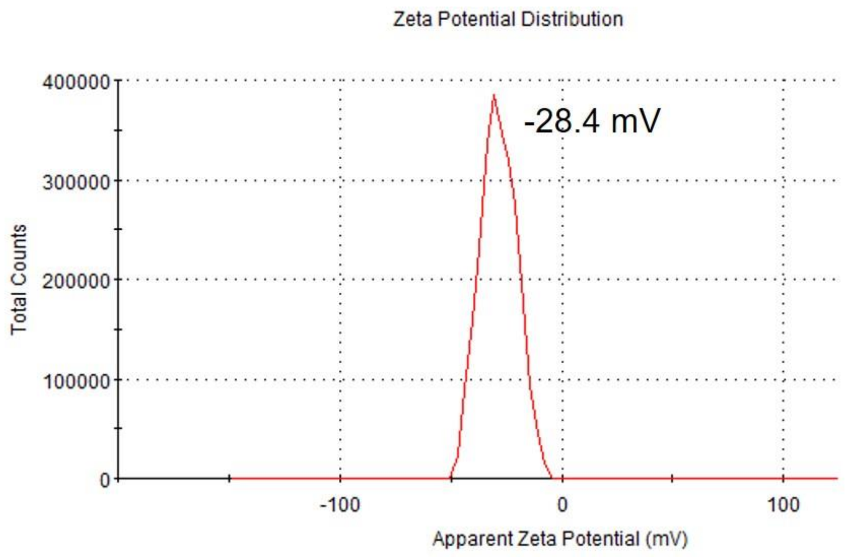

Fig. S13. Zeta potentials of CaP@BSA nanospheres in deionized water.

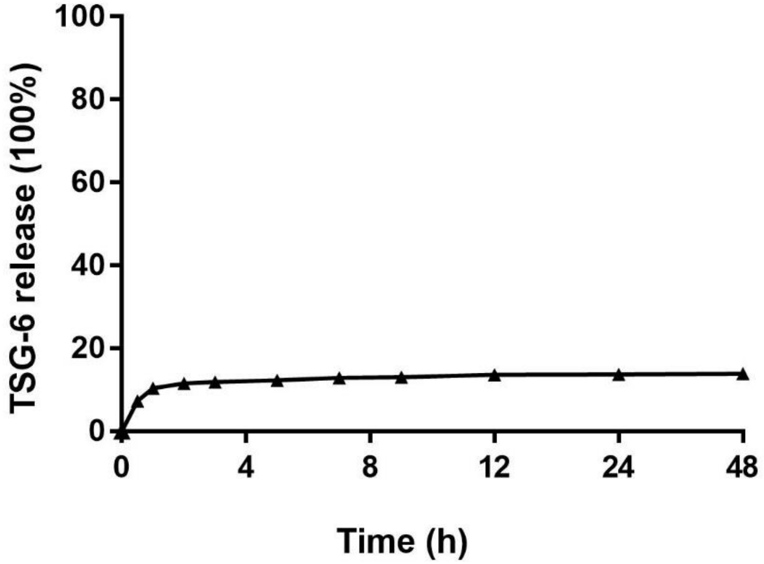

Fig. S14. Sustained release of TSG-6 by CaP@BSA nanospheres at pH 7.4.

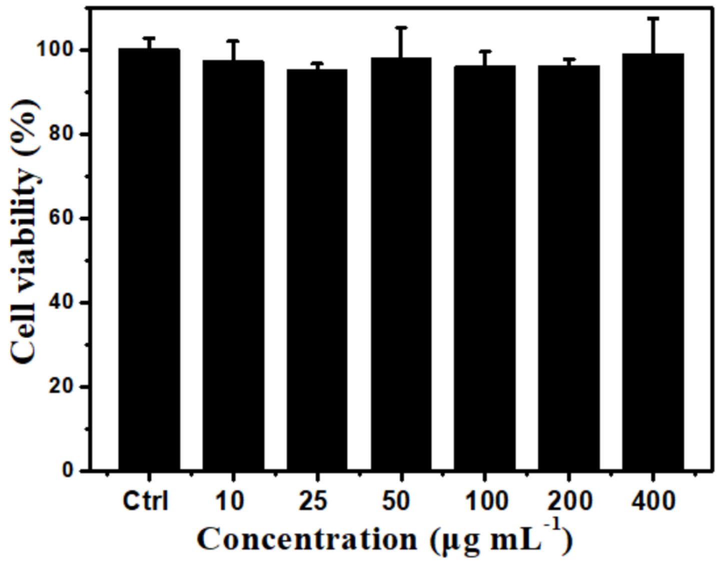

Fig. S15. Viability of MCF10a cells after 24 h of incubated with different concentrations of CaP@BSA nanospheres. Cell viability was normalized to the control group.

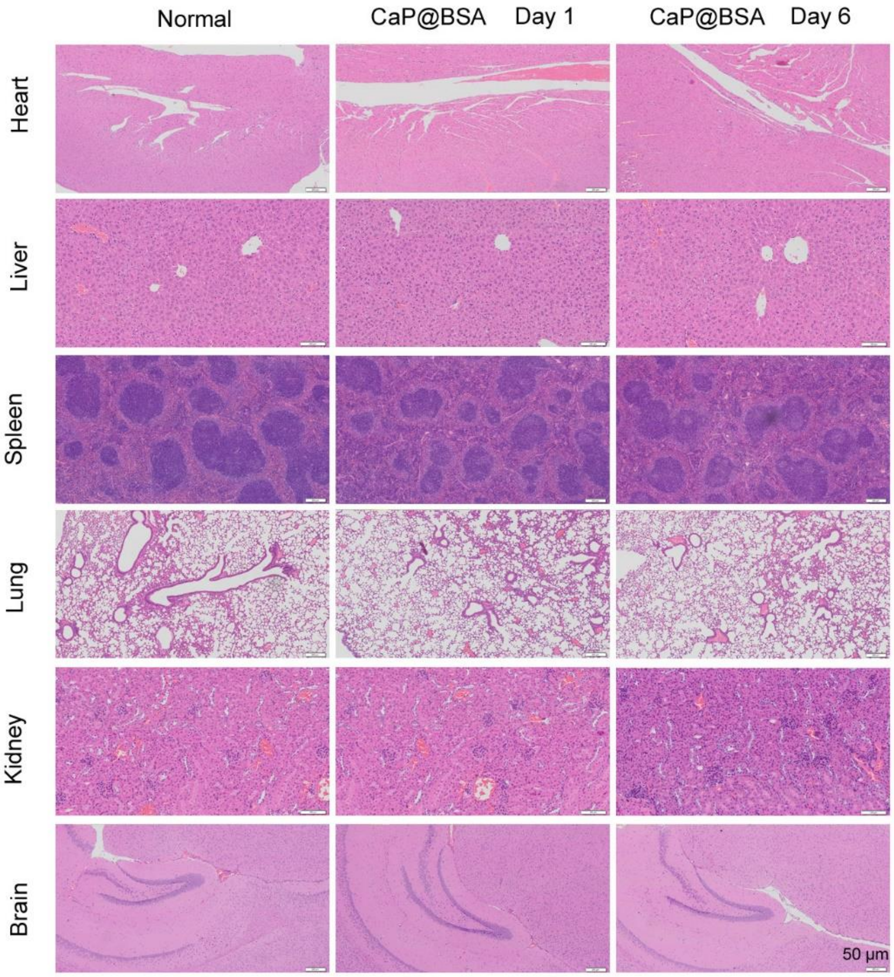

Fig. S16. Safety evaluation of CaP@BSA nanoparticles. Histopathological examination of selected organs (heart, liver, spleen, lung, kidney, and brain) from healthy mice treated with CaP@BSA nanoparticles on day 1 and day 6. No obvious organ damages were observed. n = 6.

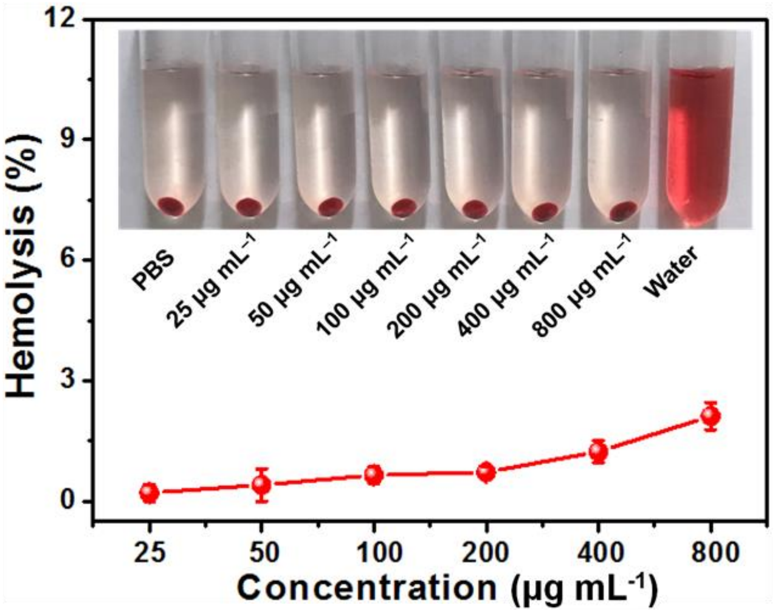

Fig. S17. Hemolysis analysis of BSA@CaP nanospheres solution at various concentrations (mean ± SD, n = 3). The mixtures after kept standing for 4 h were centrifuged to detect the hemoglobin in the supernatant visually (inset optical photographs).

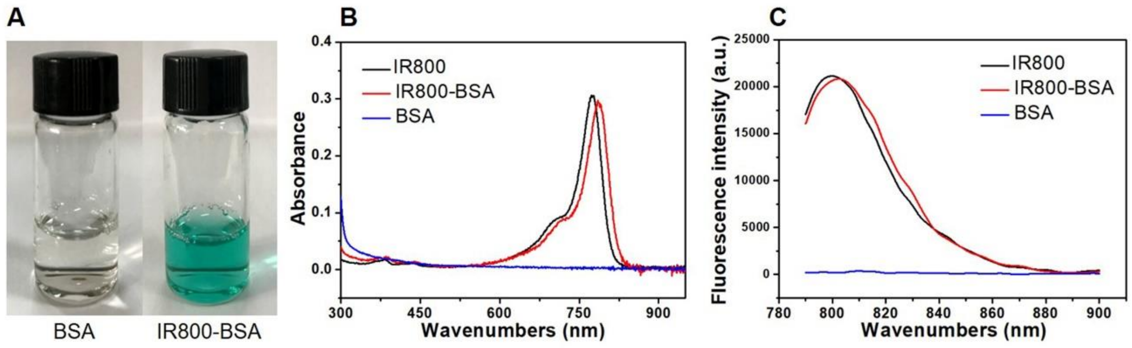

Fig. S18. The labeling of BSA by a NIR dye, IRdye 800CW NHS ester (IR800). (A) Optical photographs of the BSA and BSA-IR800 solution; (B) UV-Vis absorption of BSA and BSA-IR800 solution; (C) Fluorescence spectra of BSA and IR800-BSA solution. The results suggested the successful preparation of IR800 labeled BSA.

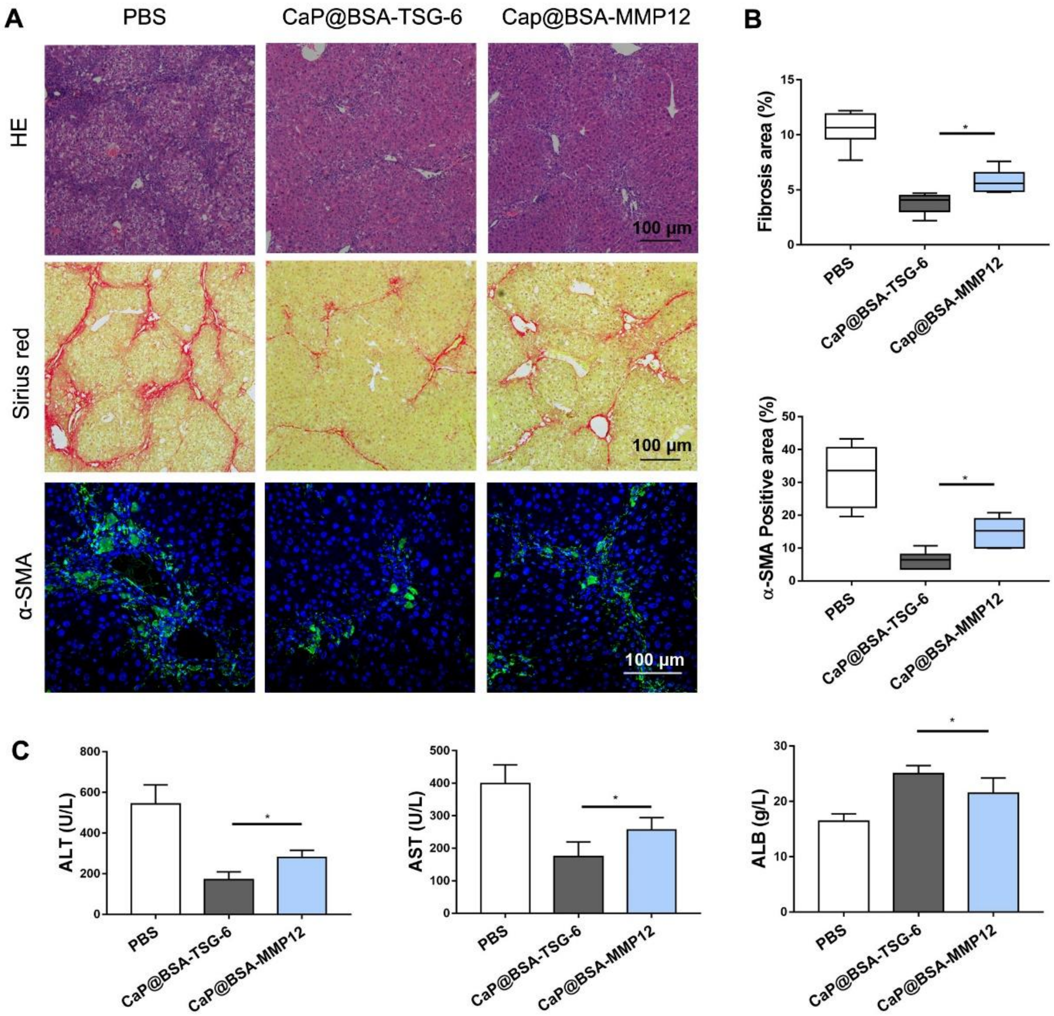

Fig. S19. CaP@BSA-MMP12 ameliorated liver fibrosis on CCL_4-induced fibrosis. (A) Representative histological images of therapeutic responses of PBS, CaP@BSA-TSG-6, and CaP@BSA-MMP12. Top, HE staining; Middle, Sirius red; Bottom α-SMA. Scale bars, 100 μm; (B) Quantification of fibrotic (Sirius red) and α-SMA^+ areas. Percentages were measured by Image-Pro Plus; C) Levels of serum ALT, AST, and ALB of indicated groups. n = 5-6. *$P < 0.05$.

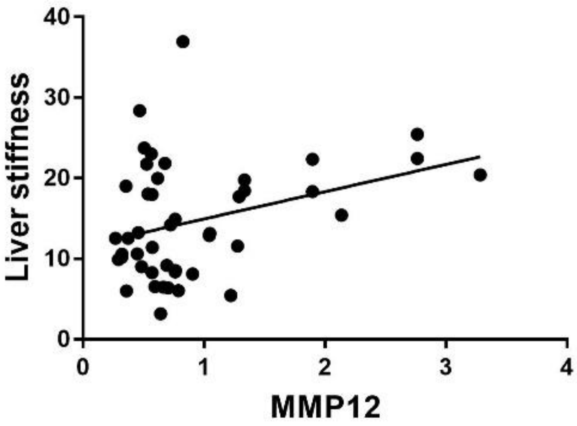

Fig. S20. Relationship of MMP12 and transient elastography (TE) in patients with primary biliary cirrhosis. TE values are expressed by liver stiffness values (Kpa). n = 50

Supplemental Table S1. Clinical information of healthy volunteers and cirrhotic patients

No.	Type	Age (year)	Sex	ALT (IU/L)	AST (IU/L)	TBIL (umol/L)	ALB (g/L)
**1**	Normal	44	Male	19	20	12	42.8
**2**	Normal	45	Male	32	25	12.6	45
**3**	Normal	49	Female	15	21	7.3	40.8
**4**	Normal	52	Female	17	17	7.2	49.3
**5**	Normal	60	Female	22	21	9.1	43.7
**6**	Cirrhosis	46	Male	72	76	48.9	26.2
**7**	Cirrhosis	51	Female	27	55	26.5	26.3
**8**	Cirrhosis	45	Male	169	48	28.7	41
**9**	Cirrhosis	55	Female	58	69	29.4	27
**10**	Cirrhosis	52	Female	43	85	33	30.9

Abbreviations: No., number; ALT, alanine aminotransferase; AST, aspartate aminotransferase; TBIL, total bilirubin; ALB, albumin.

Supplemental Table S2. Top 30 up/down-regulated transcripts after TSG-6 treatment.

Gene symbol	Fold change	Gene symbol	Fold change
**Gm10804**	39.37399771	**Mrgprf**	0.000436521
**1700046C09Rik**	33.21170614	**Sprr1b**	0.001634529
**Cpsf4l**	29.07052213	**Uts2**	0.004516782
**4930556N09Rik**	27.56419818	**Hemt1**	0.006618692
**Slc1a2**	25.67696772	**Slc7a11**	0.006961714
**Ms4a3**	22.25878248	**Lce1d**	0.010859732
**Sult3a1**	17.60472473	**Npy**	0.016495477
**B930025P03Rik**	17.00968352	**Prss56**	0.016711082
**Ctsg**	15.53016465	**S100g**	0.017320967
**Unc79**	14.5640676	**Prrt4**	0.020340893
**Ppp1r3g**	14.32570346	**Gm12603**	0.024230685
**Rgs16**	14.04957763	**Mmp13**	0.028633249
**Prok2**	13.76139371	**Fbxw14**	0.028737559
**Mpo**	13.70192584	**Gm4827**	0.03167292
**4930519G04Rik**	13.26135926	**Sprr1a**	0.03312441
**Prss34**	13.12380858	**Csn3**	0.033425228
**Cib3**	13.03751547	**Try4**	0.03424401
**Sun3**	12.89830451	**Fosl1**	0.035644547
**Ngp**	12.65407185	**Mep1b**	0.036997201
**Tas2r139**	12.48310584	**Areg**	0.04071239
**Olig1**	12.10173527	**Bmp8b**	0.041037949
**1700023A20Rik**	11.46240697	**Insm1**	0.041255318

**Ltf**	11.19201699	**Mmp12**	0.042056984
**Gm10790**	10.91925525	**Trpv6**	0.042284471
**0610033M10Rik**	10.59213829	**Prr32**	0.04393365
**Olfr728**	9.664553506	**1700016C15Rik**	0.044281092
**B230303A05Rik**	9.57485022	**Derl3**	0.046978253
**Cyp2g1**	9.481672918	**Pnlip**	0.047857248
**9130409I23Rik**	9.378009517	**Phlda2**	0.049559494
**Gm13522**	9.278024647	**Zcchc12**	0.051076479

Supplemental Table S3. Clinical information of Cirrhotic tissue microarray.

Position	No.	Age	Sex	Organ	Pathology diagnosis	Type	Tissue ID.	HBV
**A1**	1	45	M	Liver	Liver tissue	Normal	Dlv06N001	No info
**A2**	2	45	M	Liver	Liver tissue	Normal	Dlv06N001	No info
**A3**	3	36	M	Liver	Liver tissue	Normal	Dlv08N032	No info
**A4**	4	36	M	Liver	Liver tissue	Normal	Dlv08N032	No info
**A5**	5	35	F	Liver	Liver tissue	Normal	Dlv03N009	No info
**A6**	6	35	F	Liver	Liver tissue	Normal	Dlv03N009	No info
**A7**	7	43	M	Liver	Liver tissue	Normal	Dlv06N019	No info
**A8**	8	43	M	Liver	Liver tissue	Normal	Dlv06N019	No info
**A9**	9	40	M	Liver	Liver tissue	Normal	Dlv05N014	No info
**A10**	10	40	M	Liver	Liver tissue	Normal	Dlv05N014	No info
**A11**	11	27	M	Liver	Liver tissue	Normal	Dlv08N047	No info
**A12**	12	27	M	Liver	Liver tissue	Normal	Dlv08N047	No info
**A13**	13	40	M	Liver	Liver tissue	Normal	Dlv05N013	No info
**A14**	14	40	M	Liver	Liver tissue	Normal	Dlv05N013	No info
**A15**	15	23	M	Liver	Liver tissue	Normal	Dlv15N145	No info
**A16**	16	23	M	Liver	Liver tissue	Normal	Dlv15N145	No info
**B1**	17	38	M	Liver	Liver tissue	Normal	Dlv05N015	No info
**B2**	18	38	M	Liver	Liver tissue	Normal	Dlv05N015	No info
**B3**	19	16	M	Liver	Liver tissue	Normal	Dlv06N007	No info
**B4**	20	16	M	Liver	Liver tissue	Normal	Dlv06N007	No info
**B5**	21	21	F	Liver	Liver tissue	Normal	Dlv06N023	No info
**B6**	22	21	F	Liver	Liver tissue	Normal	Dlv06N023	No info
**B7**	23	50	F	Liver	Liver tissue	Normal	Dlv03N005	No info
**B8**	24	50	F	Liver	Liver tissue	Normal	Dlv03N005	No info
**B9**	25	45	M	Liver	Liver tissue	Normal	Dlv15N146	No info
**B10**	26	45	M	Liver	Liver tissue	Normal	Dlv15N146	No info
**B11**	27	27	F	Liver	Liver tissue	Normal	Dlv15N002	No info
**B12**	28	27	F	Liver	Liver tissue	Normal	Dlv15N002	No info
**B13**	29	50	M	Liver	Liver tissue	Normal	Dlv06N011	No info
**B14**	30	50	M	Liver	Liver tissue (sparse)	Normal	Dlv06N011	No info
**B15**	31	35	M	Liver	Liver tissue	Normal	Dlv05N006	No info
**B16**	32	35	M	Liver	Liver tissue	Normal	Dlv05N006	No info
**F1**	81	50	F	Liver	Nodular cirrhosis	Cirrhosis	Dlv061442	-
**F2**	82	50	F	Liver	Nodular cirrhosis	Cirrhosis	Dlv061442	-

**F3**	83	43	F	Liver	Nodular cirrhosis	Cirrhosis	Dlv021225	-
**F4**	84	43	F	Liver	Nodular cirrhosis	Cirrhosis	Dlv021225	-
**F5**	85	36	F	Liver	Nodular cirrhosis	Cirrhosis	Dlv051498	-
**F6**	86	36	F	Liver	Nodular cirrhosis	Cirrhosis	Dlv051498	-
**F7**	87	54	M	Liver	Nodular cirrhosis	Cirrhosis	Dlv024182	-
**F8**	88	54	M	Liver	Nodular cirrhosis	Cirrhosis	Dlv024182	-
**F9**	89	45	M	Liver	Nodular cirrhosis	Cirrhosis	Dlv030792	-
**F10**	90	45	M	Liver	Nodular cirrhosis	Cirrhosis	Dlv030792	-
**F11**	91	57	M	Liver	Nodular cirrhosis	Cirrhosis	Dlv021223	-
**F12**	92	57	M	Liver	Nodular cirrhosis	Cirrhosis	Dlv021223	-
**F13**	93	62	M	Liver	Nodular cirrhosis	Cirrhosis	Dlv040159	-
**F14**	94	62	M	Liver	Nodular cirrhosis	Cirrhosis	Dlv040159	-
**F15**	95	46	M	Liver	Nodular cirrhosis	Cirrhosis	Dlv022077	-
**F16**	96	46	M	Liver	Nodular cirrhosis	Cirrhosis	Dlv022077	-
**G1**	97	47	F	Liver	Nodular cirrhosis	Cirrhosis	Dlv051698	-
**G2**	98	47	F	Liver	Nodular cirrhosis	Cirrhosis	Dlv051698	-
**G3**	99	49	M	Liver	Nodular cirrhosis	Cirrhosis	Dlv031903	-
**G4**	100	49	M	Liver	Nodular cirrhosis	Cirrhosis	Dlv031903	-
**G5**	101	40	M	Liver	Nodular cirrhosis	Cirrhosis	Dlv060269	-
**G6**	102	40	M	Liver	Nodular cirrhosis	Cirrhosis	Dlv060269	-

**G7**	103	46	M	Liver	Nodular cirrhosis	Cirrhosis	Dlv024190	-
**G8**	104	46	M	Liver	Nodular cirrhosis	Cirrhosis	Dlv024190	-
**G9**	105	25	M	Liver	Nodular cirrhosis with bile duct proliferation	Cirrhosis	Dlv061381	-
**G10**	106	25	M	Liver	Nodular cirrhosis	Cirrhosis	Dlv061381	-
**G11**	107	59	M	Liver	Nodular cirrhosis	Cirrhosis	Dlv140338	-
**G12**	108	59	M	Liver	Nodular cirrhosis	Cirrhosis	Dlv140338	-
**G13**	109	53	M	Liver	Nodular cirrhosis	Cirrhosis	Dlv140341	-
**G14**	110	53	M	Liver	Nodular cirrhosis	Cirrhosis	Dlv140341	-
**G15**	111	54	M	Liver	Nodular cirrhosis	Cirrhosis	Dlv140514	-
**G16**	112	54	M	Liver	Nodular cirrhosis	Cirrhosis	Dlv140514	-
**H1**	113	46	F	Liver	Nodular cirrhosis	Cirrhosis	Dlv051285	+
**H2**	114	46	F	Liver	Nodular cirrhosis	Cirrhosis	Dlv051285	+
**H3**	115	37	M	Liver	Nodular cirrhosis	Cirrhosis	Dlv022878	+
**H4**	116	37	M	Liver	Nodular cirrhosis	Cirrhosis	Dlv022878	+
**H5**	117	58	F	Liver	Nodular cirrhosis	Cirrhosis	Dlv051047	+
**H6**	118	58	F	Liver	Nodular cirrhosis	Cirrhosis	Dlv051047	+
**H7**	119	70	M	Liver	Nodular cirrhosis	Cirrhosis	Dlv030662	+
**H8**	120	70	M	Liver	Nodular cirrhosis	Cirrhosis	Dlv030662	+
**H9**	121	38	M	Liver	Nodular cirrhosis	Cirrhosis	Dlv030419	+

**H10**	122	38	M	Liver	Nodular cirrhosis	Cirrhosis	Dlv030419	+
**H11**	123	42	M	Liver	Nodular cirrhosis	Cirrhosis	Dlv010980	+
**H12**	124	42	M	Liver	Nodular cirrhosis	Cirrhosis	Dlv010980	+
**H13**	125	43	M	Liver	Nodular cirrhosis with bile duct proliferation	Cirrhosis	Dlv030116	+
**H14**	126	43	M	Liver	Nodular cirrhosis	Cirrhosis	Dlv030116	+
**H15**	127	60	M	Liver	Nodular cirrhosis	Cirrhosis	Dlv024197	+
**H16**	128	60	M	Liver	Nodular cirrhosis	Cirrhosis	Dlv024197	+
**I1**	129	55	M	Liver	Nodular cirrhosis	Cirrhosis	Dlv060416	+
**I2**	130	55	M	Liver	Nodular cirrhosis	Cirrhosis	Dlv060416	+
**I3**	131	33	M	Liver	Nodular cirrhosis	Cirrhosis	Dlv061098	+
**I4**	132	33	M	Liver	Nodular cirrhosis	Cirrhosis	Dlv061098	+
**I5**	133	52	M	Liver	Nodular cirrhosis	Cirrhosis	Dlv060937	+
**I6**	134	52	M	Liver	Nodular cirrhosis	Cirrhosis	Dlv060937	+
**I7**	135	24	M	Liver	Nodular cirrhosis	Cirrhosis	Dlv060263	+
**I8**	136	24	M	Liver	Nodular cirrhosis with bile duct proliferation	Cirrhosis	Dlv060263	+

Abbreviations: No., number; M, Male; F, Female.

Supplemental Table S4. Clinical assessment of patients with hepatitis B related cirrhosis.

**Factors**	**Normal (n = 20)**	**Cirrhosis (n = 20)**	***P* value**
**Age (year)**	45.3 ±2.275	51.05 ±2.464	0.0945
**Female (%)**	11（55%）	8（40%）	0.5273
**ALT (IU/L)**	27.9(15-33)	36.9(18.3-46.8)	0.3329
**AST (IU/L)**	23.8(16.8-26.3)	44.2(21.5-59.8)	0.003
**TBIL (umol/L)**	14.4(11.8-17.8)	23.9(14.1-30.4)	0.0018
**ALB (g/L)**	45.7(44.5-47.4)	34(28.5-38.5)	＜0.0001

Data are expressed as the Median (25th, 75th percentiles) or N (%).

Abbreviations: n, number of subjects; ALT, alanine aminotransferase; AST, aspartate aminotransferase; TBIL, total bilirubin; ALB, albumin. Kruskal-Wallis test for continuous factors and Pearson's chi-square or Fisher's exact test for categorical factors were used.

Supplemental Table S5. Clinical assessment of patients with primary biliary cirrhosis

Factors	F1 (n = 10)	F2 (n = 10)	F3 (n = 10)	F4 (n = 10)	*P* value
**Age (year)**	55.9±6.9	56.3±5.9	48.9±9.7	58.7±9.5	0.2218
**Female (%)**	9(90%)	6(60%)	10(100%)	18(90%)	0.054
**BMI**	22.7(20.4-24.7)	23(21.7-25.3)	21.3(19.2-21.9)	22.1(20.2-23.4)	0.2112
**AMA + (%)**	10(100%)	9(90%)	9(90%)	18(90%)	1.087
**ALT (IU/L)**	27(16.5-32.3)	33.1(20-33.3)	85.7(50.8-113.5)	48.8(26.8-64)	0.0004
**AST (IU/L)**	32.2(25-31.5)	35.7(27.3-34.5)	94.3(49-117.3)	62.7(41-85.8)	0.0003
**ALP(IU/L)**	116.2(75.8-129.3)	202.6(113.5-224.3)	403(165.3-514.8)	184.5(124.3-242.5)	0.0003
**GGT(IU/L)**	106.4(31.5-106.3)	171.9(72-186.3)	455.5(130.5-733.8)	130.8(62.3-153.8)	0.0005
**TBIL (μmol/L)**	13.6(10.2-16.3)	12.34(11.1-12.4)	17(9.34-22.8)	51.4(20.8-65.6)	0.0074
**ALB (g/L)**	39.8(38.3-42.1)	38.7(37.1-40.56)	38.3(34.9-41.67)	30.8(26.9-33.7)	$<$ 0.0001
**PLT(×10^9/L)**	195.5±47.1	135±52	130.4±57.3	105.1±75.6	0.0096
**INR**	1.0±0.1	1.12±0.4	0.9±0.1	1.2±0.2	0.0256
**MRS**	1.1±0.3	1.1±0.5	1.3±0.5	3.8±1.9	0.0226

Data are expressed as the Median (25th, 75th percentiles) or N (%).

Abbreviations: n, number of subjects; BMI, body mass index; AMA, antimitochondrial antibody; ALT, alanine aminotransferase; AST, aspartate aminotransferase; ALP, alkaline phosphatase; GGT, gamma glytammyl transpeptidase; TBIL, total bilirubin; ALB, albumin; PLT, platelet; INR, international standardized ratio; MRS, Mayo risk score[7]. P values correspond to the comparison of the four subject groups. Kruskal-Wallis test for continuous factors and Pearson's chi-square or Fisher's exact test for categorical factors were used.

Supplemental Table S6. Primers used in the qRT-PCR analysis.

Gene	Forward Primer	Reverse Primer
**Mouse**		
GAPDH	TGTGTCCGTCGTGGATCTGA	TTGCTGTTGAAGTCGCAGGAG
MMP12	GATGGATGAAGCGGTACCTCACTTA	GAGTCACATCACTCCAGACTTGGAA
TNF-α	GACCCTCACACTCAGATCATCT	CCTCCACTTGGTGGTTTGCT
IL-23a	AGGACGTGTGTTGTTATTGTTCTGT	CTCTGGCGTTTGTTTCTTTTATCTT
CD163	ACTTCACAATCACTTCATGACACA	TCGTCGATTCAGACTCCTCAG
Arg1	CTCCAACCCAAAGACCTTAGTG	AGGAGCAGTCATTCGGGACTTC
CD206	CGAGCCGAGAGTAGCAGTTGTAG	AGCCATTGTCGCACACGAG
α-SMA	CGAGCCGAGAGTAGCAGTTGTAG	AGCCATTGTCGCACACGAG
Collagen I	TCTAGACATGTTCAGCTTTGTGGAC	TCTGTACGCAGGTGATTGGTG
Tropoelastin	GGTGGTATTGGTGGCATCGG	GCCTTGGCTTTGACTCCTGTG
IL-6	CCACTTCACAAGTCGGAGGCTTA	GCAAGTGCATCATCGTTGTTCATAC
IL-1β	TCCAGGATGAGGACATGAGCAC	GAACGTCACACACCAGCAGGTTA
**Human**		
β-actin	CGGTTCCGATGCCCTGAGGCTCTT	CGTCACACTTCATGATGGAATTGA
TSG-6	GAAGCTCGGGCTGGCAGATA	ACAGTTGGGCCCAGGTTTCA
MFGE8	GGGGCTTGCATCTCTTCAG	CTGCAGCCACTGATCGTTA
IL-10	GAGATGCCTTCAGCAGAGTGAAGA	AGTTCACATGCGCCTTGATGTC
HGF	CCGCTGGGAGTACTGTGCAA	AATCCCAACGCTGACATGGAA
IL-1Ra	AAGATGTGCCTGTCCTGTGTCAA	GTTCTCGCTCAGGTCAGTGATGTTA
IFN-γ	TTCAGCTCTGCATCGTTTTG	TTACTGGGATGCTCTTCGAC
COX2	CTGGAACATGGAATTACCCAGTTTG	TGGAACATTCCTACCACCAGCA
TGF-β	TCCTGGCGATACCTCAGCAA	ACATGGGCTACAGGCTTGTCACT
IDO	GAGCAGACTACAAGAATGGCACAC	GAAGCTGGCCAGACTCTATGAGAT
CX3CR1	GTGGTGCTGACAAAGCTTGGAA	TCACTGGGTGCCATCGTAAGAA

Supplemental Table S7. Antibodies used for Western blot and IHC

Antibody	Company	Catalog Number	Dilution Factor
**MMP12**	Abcam	AB52897	1:200
**α-SMA**	Sigma	A2547	1:500
**TSG-6**	RD	MAB2104	1:200
**F4/80**	Abcam	AB6640,	1:100
**iNOS**	Abcam	AB15323	1:100
**CD206**	Abcam	AB64693	1:100
**Arg1**	Abcam	AB124917	1:1000
**p-STAT3**	Abcam	AB76315	1:100000
**STAT3**	Abcam	AB119325	1:2000
**p-STAT1**	Abcam	AB30645	1:5000
**STAT1**	Abcam	AB31369	1:2000
**p-P65**	Cell Signaling	#3033	1:1000
**P65**	Cell Signaling	#8242	1:1000
**p-Akt**	Cell Signaling	#9271	1:1000
**Akt**	Cell Signaling	#2920	1:1000
**CD11b**	Abcam	AB133357	1:100
**β-actin**	Sigma	A1987	1:5000

References:

1. Wang M, Liang C, Hu H, Zhou L, Xu B, Wang X, et al. Intraperitoneal injection (IP), Intravenous injection (IV) or anal injection (AI)? Best way for mesenchymal stem cells transplantation for colitis. Sci Rep. 2016; 6: 30696.
2. Ma PF, Gao CC, Yi J, Zhao JL, Liang SQ, Zhao Y, et al. Cytotherapy with M1-polarized macrophages ameliorates liver fibrosis by modulating immune microenvironment in mice. J Hepatol. 2017; 67: 770-9.
3. Aparicio-Vergara M, Tencerova M, Morgantini C, Barreby E, Aouadi M. Isolation of Kupffer Cells and Hepatocytes from a Single Mouse Liver. Methods Mol Biol. 2017; 1639: 161-71.
4. Hu H, Yin J, Wang M, Liang C, Song H, Wang J, et al. GX1 targeting delivery of rmhTNFalpha evaluated using multimodality imaging. Int J Pharm. 2014; 461: 181-91.
5. Cohen R, Vugts DJ, Stigter-van Walsum M, Visser GW, van Dongen GA. Inert coupling of IRDye800CW and zirconium-89 to monoclonal antibodies for single- or dual-mode fluorescence and PET imaging. Nat Protoc. 2013; 8: 1010-8.
6. You Q, Holt M, Yin H, Li G, Hu CJ, Ju C. Role of hepatic resident and infiltrating macrophages in liver repair after acute injury. Biochem Pharmacol. 2013; 86: 836-43.
7. Corpechot C, Carrat F, Poujol-Robert A, Gaouar F, Wendum D, Chazouilleres O, et al. Noninvasive elastography-based assessment of liver fibrosis progression and prognosis in primary biliary cirrhosis. Hepatology. 2012; 56: 198-208.
